# Exome-wide somatic mutation characterization of small bowel adenocarcinoma

**DOI:** 10.1371/journal.pgen.1007200

**Published:** 2018-03-09

**Authors:** Ulrika A. Hänninen, Riku Katainen, Tomas Tanskanen, Roosa-Maria Plaketti, Riku Laine, Jiri Hamberg, Ari Ristimäki, Eero Pukkala, Minna Taipale, Jukka-Pekka Mecklin, Linda M. Forsström, Esa Pitkänen, Kimmo Palin, Niko Välimäki, Netta Mäkinen, Lauri A. Aaltonen

**Affiliations:** 1 Genome-Scale Biology Research Program, Research Programs Unit, University of Helsinki, Helsinki, Finland; 2 Department of Medical and Clinical Genetics, Medicum, University of Helsinki, Helsinki, Finland; 3 Department of Pathology, HUSLAB, Helsinki University Hospital and University of Helsinki, Helsinki, Finland; 4 Finnish Cancer Registry, Institute for Statistical and Epidemiological Cancer Research, Helsinki, Finland; 5 Faculty of Social Sciences, University of Tampere, Tampere, Finland; 6 Department of Medical Biochemistry and Biophysics, Karolinska Institutet, Stockholm, Sweden; 7 Department of Surgery, Jyväskylä Central Hospital, Jyväskylä, Finland; 8 Faculty of Sport and Health Sciences, University of Jyväskylä, Jyväskylä, Finland; Dana Farber Cancer Institute, UNITED STATES

## Abstract

Small bowel adenocarcinoma (SBA) is an aggressive disease with limited treatment options. Despite previous studies, its molecular genetic background has remained somewhat elusive. To comprehensively characterize the mutational landscape of this tumor type, and to identify possible targets of treatment, we conducted the first large exome sequencing study on a population-based set of SBA samples from all three small bowel segments. Archival tissue from 106 primary tumors with appropriate clinical information were available for exome sequencing from a patient series consisting of a majority of confirmed SBA cases diagnosed in Finland between the years 2003–2011. Paired-end exome sequencing was performed using Illumina HiSeq 4000, and OncodriveFML was used to identify driver genes from the exome data. We also defined frequently affected cancer signalling pathways and performed the first extensive allelic imbalance (AI) analysis in SBA. Exome data analysis revealed significantly mutated genes previously linked to SBA (*TP53*, *KRAS*, *APC*, *SMAD4*, and *BRAF*), recently reported potential driver genes (*SOX9*, *ATM*, and *ARID2*), as well as novel candidate driver genes, such as *ACVR2A*, *ACVR1B*, *BRCA2*, and *SMARCA4*. We also identified clear mutation hotspot patterns in *ERBB2* and *BRAF*. No *BRAF* V600E mutations were observed. Additionally, we present a comprehensive mutation signature analysis of SBA, highlighting established signatures 1A, 6, and 17, as well as U2 which is a previously unvalidated signature. Finally, comparison of the three small bowel segments revealed differences in tumor characteristics. This comprehensive work unveils the mutational landscape and most frequently affected genes and pathways in SBA, providing potential therapeutic targets, and novel and more thorough insights into the genetic background of this tumor type.

## Author summary

Small bowel adenocarcinoma is a rare but aggressive disease with limited treatment options. Of gastrointestinal tumors, small bowel tumors account for 3%, of which around one third are adenocarcinomas. Due to the scarcity of evidence-based treatment recommendations there is a dire need for knowledge on the biology of these tumors. Here, we performed the first large exome sequencing effort of 106 small bowel adenocarcinomas from a Finnish population-based cohort to comprehensively characterize the genetic background of this tumor type. The set included tumors from all three small bowel segments allowing us to also compare the genetic differences between these subsets. We defined significantly mutated genes and frequently affected pathways, providing potential therapeutic targets, such as *BRAF*, *ERBB2*, *ERBB3*, *ERBB4*, *PIK3CA*, *KRAS*, *ATM*, *ACVR2A*, *ACVR1B*, *BRCA2*, and *SMARCA4*, for this disease.

## Introduction

The gastrointestinal tract, a continuous passageway, includes the main digestive organs: the stomach, the small bowel, and the large bowel. The small bowel makes up 75% of the length of the gastrointestinal tract, yet small bowel tumors constitute only approximately 3% of gastrointestinal tumors [1]. The major histological types of primary small bowel cancers are carcinoids, adenocarcinomas, lymphomas, and sarcomas. Small bowel adenocarcinomas (SBAs) account for around one third of the tumors and are most often found in the duodenum, the first section of the small bowel [2].

SBAs are often sporadic, however, several factors such as inflammatory bowel disease (IBD; Crohn's disease and ulcerative colitis) and hereditary syndromes such as familial adenomatous polyposis (FAP) and Lynch syndrome (LS) are known to predispose to these tumors [3]. Patients with celiac disease are also at a greater risk of developing SBA compared to general population. Other risk determinants include lifestyle factors, such as alcohol use, obesity, and consumption of red meat [4].

Although diagnostic tools such as imaging and endoscopy have improved, SBAs are often advanced at the time of diagnosis and sometimes found incidentally. The estimated five-year relative survival rate for SBA is 40%, indicating a worse prognosis than for colorectal adenocarcinomas (hereinafter referred as CRC) [2]. The incidence of SBA has also increased over the past decades. This combined with the scarcity of evidence-based treatment recommendations underlines a dire need for knowledge on the biology of these tumors.

To date, there have been relatively few large studies on SBA that have either screened a set of known mutation hotspots or cancer genes [5–7], along with two exome sequencing efforts on small sets of duodenal adenocarcinomas [8,9]. The most commonly mutated genes in SBA include *TP53*, *KRAS*, *SMAD4*, and *APC* [3,7]. The fraction of microsatellite unstable (MSI) tumors in SBA has been reported to vary between 5–35% [10]. These tumors have a defective DNA mismatch repair (MMR) system and thus, compared to microsatellite stable (MSS) tumors, exhibit a remarkably high mutation burden.

SBAs share many of the above-mentioned features with CRC. They also share similar carcinogenic pathways; e.g. they are thought to arise through an adenoma-to-carcinoma transition [11]. Regardless, large bowel tumors are much more frequent. Factors that could contribute to the difference include protective factors of the small bowel environment. Due to alkalinity, fewer bacteria, liquid nature of small bowel contents, and shorter transit time, there is less exposure to carcinogens [3]. The difference in cancer incidence between the small and large bowel could also be related to a slower rate of stem cell divisions in the small bowel [12].

Since there are limited data available to guide treatment decisions, our aim was to characterize the somatic mutational landscape of SBAs using exome sequencing to gain new insights into the SBA biology and identify potential therapeutic targets.

## Results

### Cohort characteristics

Clinicopathologic features of the 106 SBA patients are listed in Table 1. Of the 106 tumors, 26 (25%) were duodenal, 52 (49%) jejunal, 18 (17%) ileal, and 10 (9.4%) resided in an unspecified location. The male-to-female ratio was 1.1, and the median age at diagnosis 62 years (range, 24 to 86 years). Median age at diagnosis was lowest for patients with jejunal tumor (59.5 years for jejunum versus 71.0 for duodenum and 63.0 for ileum; *P* = 0.00108, Kruskal-Wallis test). Fifteen tumors were designated as MSI based on the exome sequencing data (see below).

**Table 1:** Clinicopathologic features of the patient cohort.

Characteristic	No. (%) of patients
**All**	106
**Sex**	
*Male	56 (53%)
*Female	50 (47%)
**Age**	
*Median	62 years
*Range	24–86 years
**Celiac disease**	
*Celiac	10 (9.4%)
*Non-celiac	96 (91%)
**Inflammatory bowel disease**	
*Crohn’s disease	4 (3.8%)
*Ulcerative colitis	1 (0.9%)
*no inflammatory disease	101 (95.3%)
**Hereditary syndromes**	
*Lynch syndrome	4 (3.8%)
*FAP	2 (1.9%)
*no hereditary syndrome	100 (94.3%)
**Primary tumor location**	
*Duodenum	26 (24.5%)
*Jejunum	52 (49.1%)
*Ileum	18 (17.0%)
*not specified	10 (9.4%)
**Tumor stage (TNM)**	
*I	4 (3.8%)
*II	22 (20.7%)
*III	25 (23.6%)
*IV	41 (38.7%)
*not specified	14 (13.2%)
**Histological grade**	
*G1	18 (17.0%)
*G2	60 (56.6%)
*G3	20 (18.9%)
*not specified	8 (7.5%)
**MMR status**	
*MSI	15 (14.2%)
*MSS	91 (85.8%)

Ten patients in the cohort had been diagnosed with celiac disease, five with IBD, and six with hereditary syndromes (LS or FAP). The causative germline mutations in LS patients occurred in *MLH1* or *MSH6*, and in FAP patients in *APC*. All tumors from patients with IBD were MSS, whereas all LS-associated tumors were MSI, and the tumors from the two FAP patients were either MSS or MSI. Evaluation of the clinicopathologic characteristics revealed enrichment of celiac patients amongst the SBA patients: 9.4% compared to 2.4% in the general Finnish population (*P* = 2.48x10^-4^, exact binomial test) [13]. Five of 10 tumors from patients with celiac disease were microsatellite-unstable, and thus celiac disease was associated with MSI (odds ratio (OR), 8.31; 95% confidence interval (CI), 1.62–43.6; *P* = 4.83x10^-3^), which corresponds to previous literature [14]. None of the tumors related to celiac disease resided in ileum. Otherwise the celiac disease-related tumors did not notably differ from other tumors in terms of the characteristics in Table 1. Disease-specific survival was superior for patients with microsatellite-unstable tumors after adjustment for sex, tumor stage, and age at diagnosis (hazard ratio (HR), 0.111; 95% CI, 0.0292–0.419; *P* = 1.20x10^-3^) (Table a in S1 Table; S1 Fig). Also, male patients had a worse disease-specific survival, although the difference was not formally significant.

### Frequently mutated genes in exome data

Exome sequencing analysis identified 75,993 somatic mutations across all samples. Of these, 29,120 were non-synonymous and 9,415 synonymous (S2 Table). Fifteen out of 106 (14%) samples were classified as MSI based on high mutation load and overrepresentation of insertions and deletions (indels) at microsatellite loci obtained from Hause *et al*. [15]. The classification was confirmed by signature analysis (see methods). The average mutation burden in the whole target region was 4.30 mutations per megabase (mut/Mb) per MSS and 63.6 mut/Mb per MSI sample (S2 Fig). The median number of non-synonymous mutations per sample was 88 in MSS (interquartile range (IQR), 64.5–114) and 1,266 in MSI tumors (IQR, 666–1,738). The median number of missense mutations was 79 (IQR, 56.5–105) in MSS and 812 (IQR, 518–1,209) in MSI tumors. For nonsense mutations, the median mutation counts were 10 (IQR, 6.5–15) in MSS and 429 (IQR, 210–498) in MSI tumors and for frameshift mutations 4 (IQR, 2–6) in MSS and 286 (IQR, 180–397) in MSI tumors. In MSS tumors, 6,214 genes harbored a non-synonymous mutation in at least one tumor and 1,921 genes in two or more tumors as compared to 10,716 and 5,055 in MSI tumors, respectively.

In MSS tumors, the most frequently mutated known cancer genes were *TP53* (44/91, 48%), *KRAS* (43/91, 47%), *APC* (20/91, 22%), *SMAD4* (14/91, 15%), *SOX9* (11/91, 12%), *BRAF* (10/91, 11%), and *ERBB2* (10/91, 11%). In MSI tumors, among the most frequently mutated genes were known driver genes *ACVR2A* (13/15, 87%), *BMPR2* (9/15, 60%), *KRAS* (8/15, 53%), and *APC* (7/15, 47%). *TP53*, the most frequently mutated gene in MSS tumors, was also frequently mutated (6/15, 40%) in MSI tumors.

### Significantly mutated genes in SBAs

Next, we sought to identify genes showing statistical evidence of positive selection for mutations in SBA. We applied OncodriveFML to detect candidate driver genes in MSS tumors. In total, 44 genes displayed a nominally significant *P*-value (<0.05) (Table a in S3 Table). Seven genes remained significant after correction for multiple testing (false discovery rate (FDR), q-value <0.1). However, genes with *P*<0.05 were also considered as being of potential interest.

**Fig 1:**
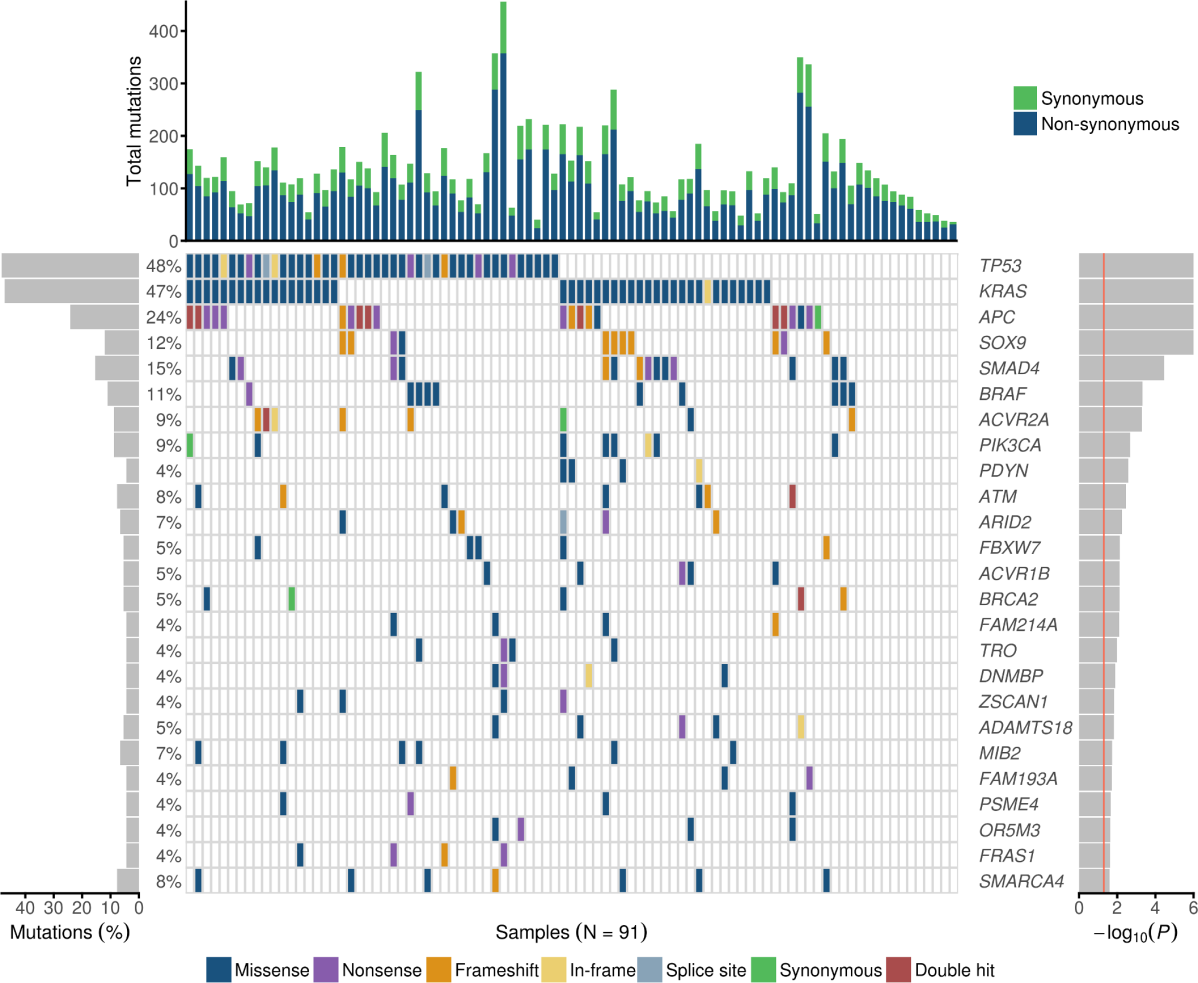
Mutational landscape of the most significant genes in MSS SBAs. The figure includes the 25 highest-ranking genes in MSS tumors (n = 91) according to OncodriveFML, ranked by the *P*-value (right, red line at *P* = 0.05). Of these, *TP53*, *KRAS*, *APC*, *SOX9*, *SMAD4*, *BRAF*, and *ACVR2A* were significant also after correction for multiple testing. Different colors distinguish between the different types of mutations (in the middle). “Double hit” refers to two truncating mutations. The percentage of mutated tumors by gene are shown on the left. The upper bars represent the total number of both synonymous and non-synonymous mutations per tumor.

The most significant genes in MSS tumors consisted of known cancer genes such as *TP53*, *KRAS*, *APC*, *SOX9*, *SMAD4*, *BRAF*, and *ACVR2A*. (Fig 1, Table a in S3 Table). The twenty-five highest-ranking driver candidates included also recently reported (*ATM* and *ARID2*) and novel candidate drivers such as *ACVR1B*, *BRCA2*, and *SMARCA4* that (to our knowledge) have not been implicated in SBA before. More information on the mutation content of the genes (*P*<0.05) is displayed in Table b in S3 Table.

In addition to *KRAS*, *APC* was designated as one of the most significant genes in MSS tumors (20/91, 22%) and was also frequently mutated in MSI (7/15, 46.7%) tumors. Of note, 37 of 42 (88%) *APC* mutations were protein-truncating (22 nonsense and 15 frameshift). Of the five patients with IBD, two (40%) harbored an *APC* nonsense mutation.

### Atypical mutation hotspots of *BRAF*

**Fig 2:**
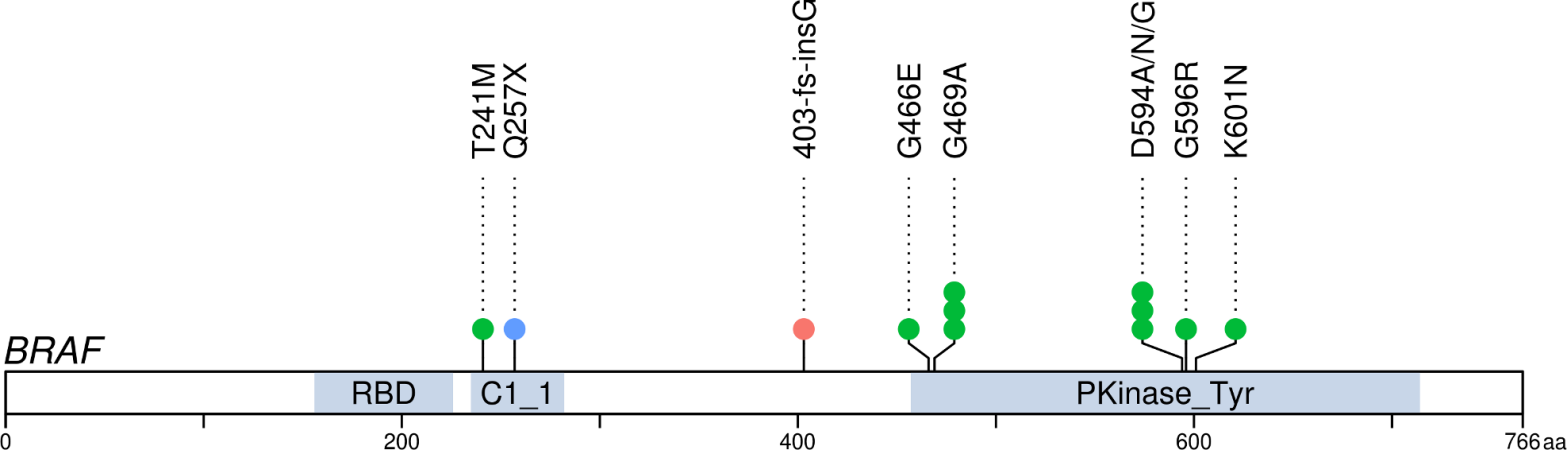
Mutations in *BRAF* (ENST00000288602). In total, 12 mutations were identified in 11 tumors (MSS n = 10, MSI n = 1). RBD = Raf-like Ras-binding domain; C1_1 = C1 domain; Pkinase_Tyr = Protein tyrosine kinase.

*BRAF* was mutated in 11 tumors (11/106, 10.4%): 10 MSS and one MSI (Fig 2). We did not observe any V600E mutations. Instead, we identified an atypical mutation pattern with two known, less studied hotspots: G469A with two and D594A/G/N with three hits. In addition, we observed other known mutations near these hotspots (G466E, G596R, and K601N). All above-mentioned mutations resided in exons 11 or 15 and have been designated as somatic hotspots in various cancers [16]. In read level inspection, we identified one additional tumor (SIA56) displaying a hotspot mutation in G469A supported by four mutant reads which had not been called. This tumor also harbored one missense mutation in *BRAF* (T241M). Furthermore, two tumors harbored protein-truncating *BRAF* variants: Q257X (SIA214) and A404fs (SIA53). Except for one frameshift mutation, all other mutations occurred in MSS tumors. We compared tumor and patient characteristics according to *BRAF* mutation status, no significant differences were detected (Table a in S4 Table).

*BRAF* V600E and *KRAS* mutations are generally mutually exclusive. Regarding atypical hotspot mutations, however, we identified four out of 11 *BRAF* mutants where *BRAF* and *KRAS* mutations co-occurred: *KRAS*^A146T^+*BRAF*^D594A^ (SIA121), *KRAS*^G12R^+*BRAF*^G469A^ (SIA228), *KRAS*^G12D^+*BRAF*^Q257X^ (SIA214), and *KRAS*^G12D^+*BRAF*^A404fs^ (SIA53).

### Mutation patterns of *ERBB2* and other ERBB receptor family member genes

**Fig 3:**
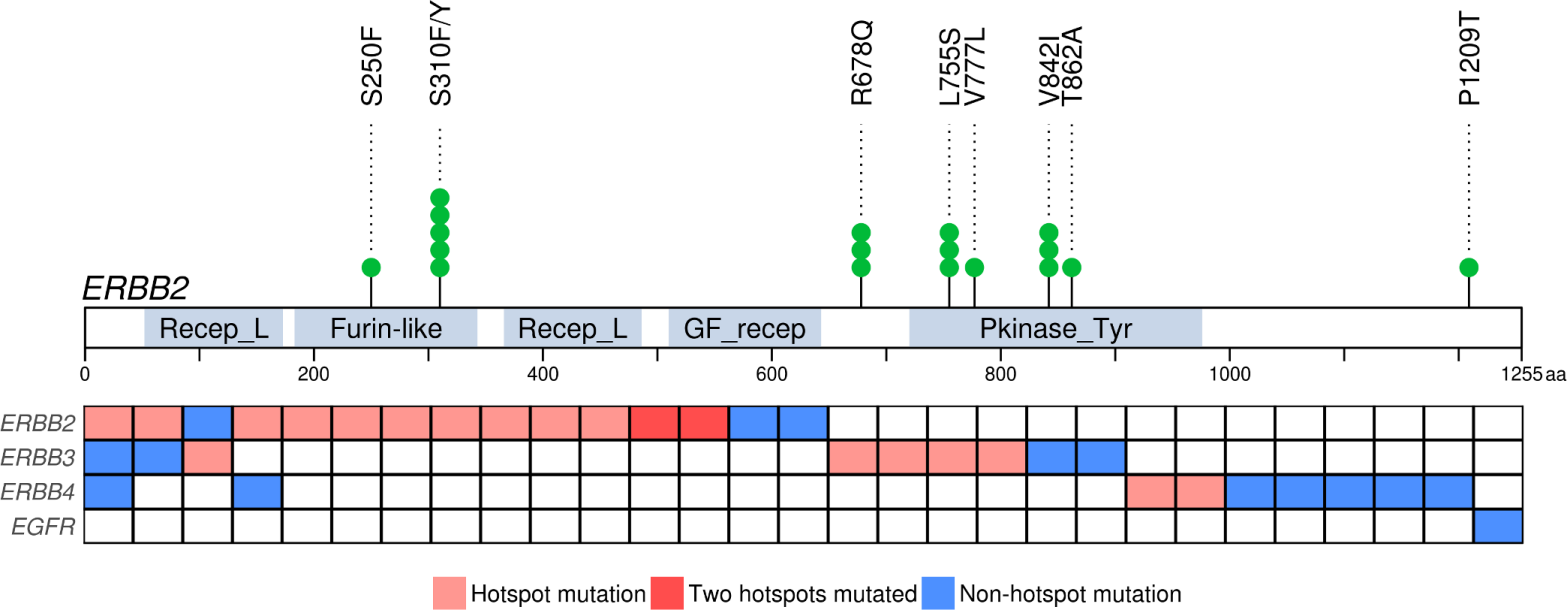
Mutation pattern in ERBB receptor family. Mutations in *ERBB2* (ENST00000269571) grouped into four hotspots (top). Samples (n = 29) with a mutated member of ERBB receptor family are presented in columns (below). In addition to a hotspot mutation, some samples displayed simultaneously a non-hotspot mutation in the same gene, thus all mutations are not shown in the figure. Recep_L = Receptor L domain; Furin-like = Furin-like cysteine rich region; GF_recep = Growth factor receptor domain; Pkinase_Tyr = Protein tyrosine kinase.

We identified 18 *ERBB2* mutations in 15 tumors (15/106, 14%): 10 MSS and five MSI (Fig 3). *ERBB2* did not reach significance in the OncodriveFML analysis; however, it is a known therapeutic target frequently mutated in many tumors of the digestive system, including those of the small bowel [5,7,17,18]. The majority (14/18, 78%) of the mutations clustered into four known hotspots (Fig 3) [16].

One of the hotspots, L755S, was mutated exclusively in MSI tumors, whereas the other hotspots, S310F/Y, R678Q, and V842I, were found both in MSS and MSI tumors. Two samples harbored concurrent hotspot mutations, L755S+V842I and R678Q+V842I. Such co-occurrence has been reported previously at least once in SBA [5]. In addition to the hotspot mutations, three single mutations were identified in MSS (S250F, V777L, and T862A) and one in MSI tumors (P1209T).

We compared tumor and patient characteristics of *ERBB2* mutant and wild-type cases (Table b in S4 Table). We detected a statistically significant difference in the MMR status (OR, 3.98; 95% CI, 0.886–16.4; *P* = 0.0368), *ERBB2* mutation frequency being higher in MSI tumors.

The ERBB family comprises of four receptor tyrosine kinases encoded by *EGFR* (also known as *ERBB1*), *ERBB2*, *ERBB3*, and *ERBB4*. Albeit with lower frequencies, also *ERBB3* and *ERBB4* displayed hotspot mutations in our data. We identified 10 *ERBB3* mutations in nine tumors, revealing two hotspots: V104M/L in one MSS and in two MSI and S846I in two MSS tumors. These affected either the extracellular domain (V104M/L) or the kinase domain (S846I). We also observed 10 *ERBB4* mutations in nine tumors. *ERBB4* displayed one mutation hotspot, L798R/P in the protein tyrosine kinase domain, supported by two MSS tumors. Moreover, we detected one *EGFR* mutation (R977C). Thus, there were altogether 29 samples (27%) with a mutation in at least one of the *ERBB* genes (Fig 3). Of these, four tumors exhibited mutations in more than one of these three genes. All hotspot mutations in different *ERBB* genes were mutually exclusive.

### Allelic imbalance in SBA

**Fig 4:**
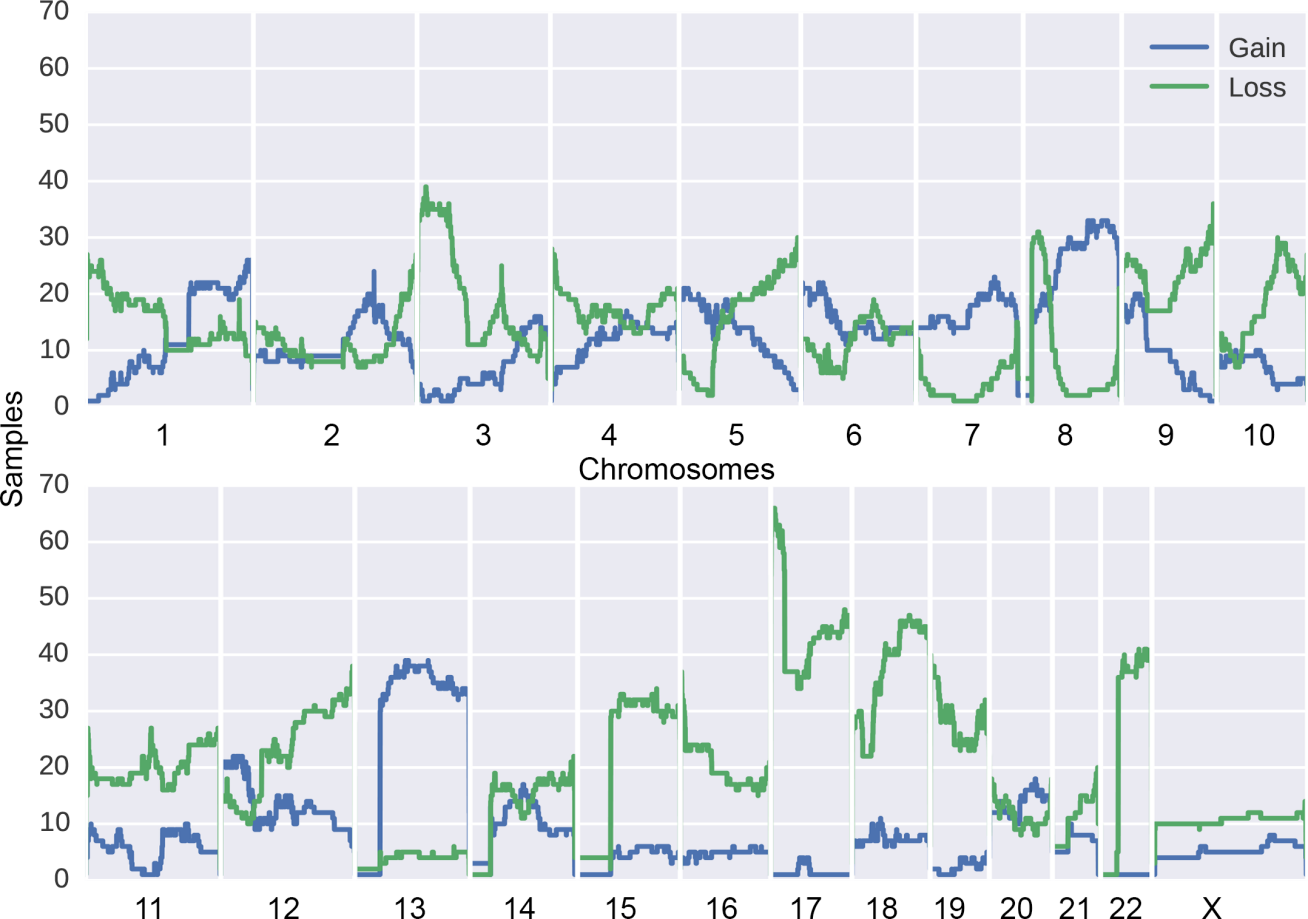
Overview of AI events in SBA. Frequency of gains and losses in 106 SBA samples.

We performed an allelic imbalance (AI) analysis for the whole data set of 106 tumors. The analysis revealed 1,541 loss and 840 gain events across all samples. The number of AI events in MSI tumors (median, 5; IQR, 4–8) was significantly lower compared to that of MSS tumors (median, 22; IQR, 13–35) (*P* = 1.95x10^-9^), see Table b in S1 Table. The number of AI events did not differ significantly between tumors from different small bowel segments. The most frequent AI event was partial or whole loss of chromosome 17 short arm (p) harboring *TP53*, detected in 62/106 (58.5%) samples (Fig 4; S3 Fig). Non-synonymous variants in *TP53* co-occurred with loss events in 41/50 (82.0%) of mutated cases (OR, 7.43; 95% CI, 2.86–21.1, P = 4.02x10^-6^) (S4 Fig). We also observed a high frequency of chromosomal losses in two other significantly mutated known cancer genes: *SMAD4* (n = 46) and *SOX9* (n = 44). Chromosome or arm level losses were observed at high frequency (n>30) at chromosomes 3p, 8p, 9q, 12q, 15, 17, 18q, 19, and 22 (Fig 4; S3 Fig).

Gain events were observed at high frequency at chromosomes 13 and 8q (with *MYC* as a possible target). In addition, known oncogenes, such as *KRAS*, *BRAF*, and *PIK3CA* that were amongst the highest-ranking genes, were clearly amplified in 20/106 (18.9%), 19/106 (17.9%), and 16/106 (15.1%) samples, respectively. We observed also localized and strong amplification at the *ERBB2* locus in 4 samples, two of which had a hotspot mutation in *ERBB2* (S3–S5 Figs).

### Mutational signatures

**Fig 5:**
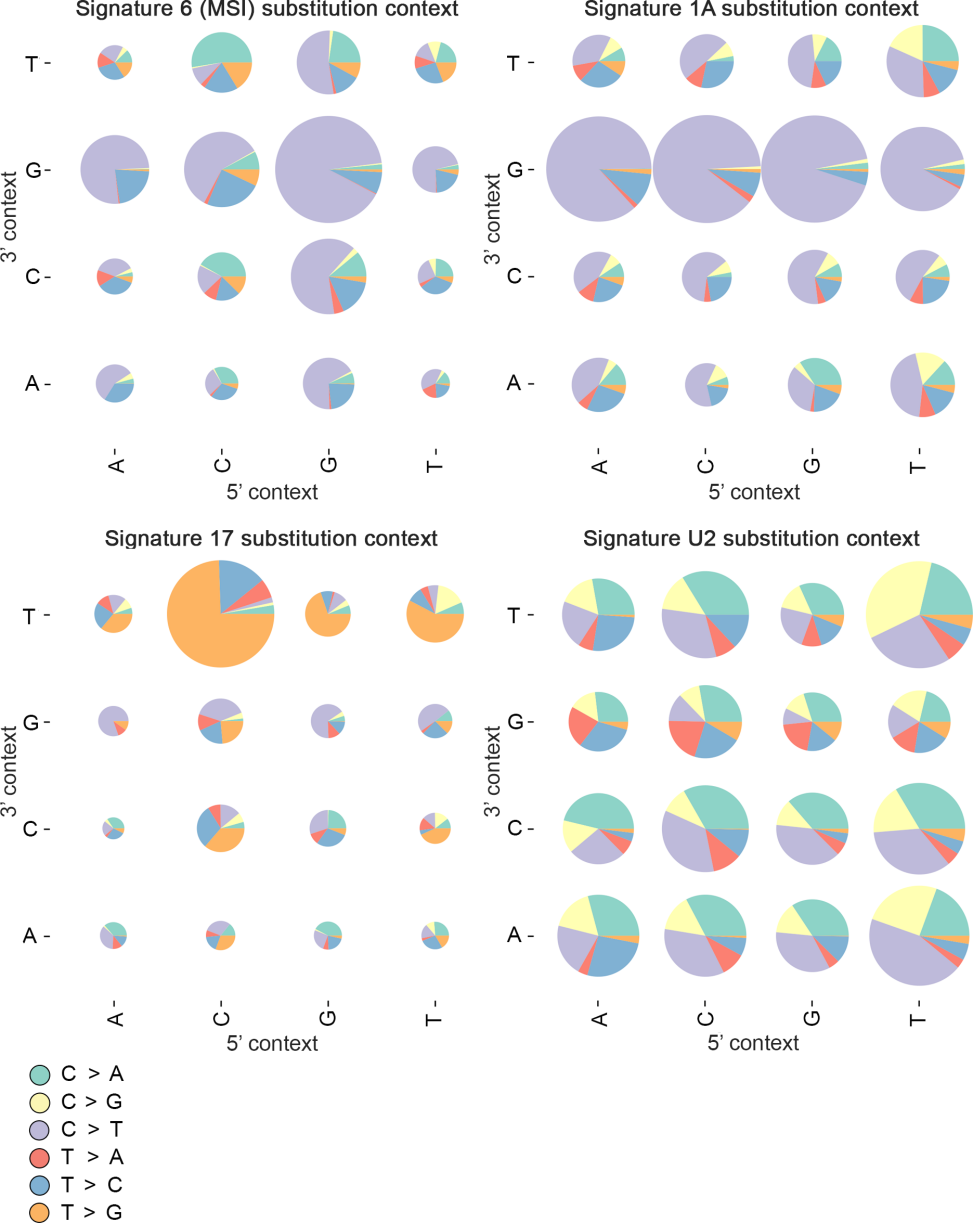
Signature contexts. The 15 MSI tumors displayed signature 6. There were three signatures (1A, 17, and U2) that could be extracted from the 91 MSS tumors.

First, we performed mutational signature analysis for all 106 samples. A known MSI signature (signature 6) was identified in 15 tumors (Fig 5; Table a in S5 Table). The signature analysis was then performed separately for the 91 MSS SBAs. This process yielded three mutational signatures (1A, 17 and U2) corresponding to known signatures reported by Alexandrov *et al*. (Fig 5; Tables b and c in S5 Table) [19]. Signature U2 has not been validated previously due to lack of available biological samples and access to BAM files for the samples. We were able to inspect read sequences in our data set and validate mutations in this signature class.

Mutational signatures were studied using multivariable-adjusted negative binomial regression (Table c in S1 Table). Similar to other cancers, the frequency of mutations attributable to signature 1A increased with age at diagnosis (increase per 10 years, 20%; 95% CI, 10–32%; *P* = 4.32x10^-5^) [19]. Exposure to signature 1A was highest in jejunal tumors; compared to duodenal tumors, there was an increase of 66% in expected mutation count (95% CI, 29%-110%; *P* = 7.17x10^-5^; see S6 Fig). No notable difference in signature 1A was observed between ileal and duodenal tumors (*P* = 0.348). Also, tumors from female patients showed an increase of 26% in the number of mutations attributable to signature 1A (95% CI, 2.9%-54%; *P* = 0.0259).

### Altered pathways in SBAs

We characterized most frequently affected cancer signalling pathways in MSS SBA, focusing on mutations in known pathways—Wnt/β-catenin, TGF-β, PI3K/AKT, ERBB, ERK/MAPK, and p53 signalling (S6 Table). The most frequently mutated pathway was PI3K/AKT, where at least one gene was mutated in the majority of tumors (77/91, 84.6%). This pathway includes the two most frequently mutated genes, *KRAS* and *TP53*. The PI3K/AKT pathway was followed by ERBB (73/91, 80.2%), ERK/MAPK (72/91, 79.1%), and Wnt/β-catenin (70/91, 76.9%) signalling pathways. Also, TGF-β and p53 signalling were affected in many tumors (66/91 (72.5%) and 63/91 (69.2%), respectively). Eighty-four out of 91 tumors (92.3%) harbored at least one non-synonymous mutation in one of these six known cancer pathways.

### Comparison of the three segments of the small bowel

We compared tumor characteristics between the three small bowel segments. Although the tumors displayed rather similar numbers of mutations, mutated known cancer genes, and MSI frequencies (S7 Table), some differences existed. In MSS tumors, the *APC* mutation frequency varied between segments; it was the lowest in jejunal tumors (13.6%), followed by ileal (31.3%) and duodenal tumors (37.5%). The *TP53* mutation frequency was lower in duodenum (29.2%) than other segments: jejunum (56.8%) and ileum (56.3%).

The differences between the segments were also reflected in the frequencies at which major signalling pathways were mutated. In duodenal tumors, the most frequently affected pathway was ERBB signalling (20/24, 83.3%). Whereas, both in jejunal and ileal tumors the most frequently affected pathway was PI3K/AKT (42/44, 95.5% and 12/16, 75.0%, respectively). The most notable differences between segments were seen in ERBB signalling which was less frequently mutated in ileal tumors (9/16, 56.3%) compared duodenal and jejunal tumors (20/24, 83.3% and 38/44, 86.4%, respectively) (*P* = 0.0463) and in ERK/MAPK signalling, most frequently affected in jejunal tumors (40/44, 90.9%) compared to duodenal and ileal tumors (18/24, 75.0% and 9/16, 56.3%, respectively) (*P* = 9.06x10^-3^).

## Discussion

Through large-scale utilization of archival tissue from nationwide population-based material, we conducted a comprehensive study of the somatic mutational landscape of primary SBA including all three small bowel segments. To our knowledge, this is the largest exome sequencing study on SBA to date. Most MSS tumors had a mutational burden of <10 mut/Mb. The median mutational burden in the whole SBA set was 3.96 mut/Mb which is in agreement with previously published results on SBA and corresponds to the mutation rates reported in CRC and gastric cancer [7,20].

The assessment of relevant genes in SBA indicated that *TP53* and *KRAS* were the most significantly mutated genes in MSS tumors, the mutation frequencies corresponding to previous reports [5–7]. The high frequency of losses in *TP53* and gains in *KRAS* provided further support for these observations. Thus, our results strengthen the pivotal roles of these genes in SBA genesis. Of note, *KRAS* was also frequently mutated in MSI tumors and its mutation status holds therapeutic value, since tumors with mutant *KRAS* do not respond to EGFR inhibitors [21]. Interestingly, *TP53* mutation frequency in the duodenum was lower than in other regions of the small bowel, a similar trend as reported by Laforest *et al*. [5].

*APC* reached an equally high level of significance with *TP53* and *KRAS* in the analysis of MSS tumors. The role of mutant *APC* in the pathogenesis of SBA has been under debate. Some have proposed, in contrast to colorectal carcinogenesis, that *APC* would not play such an essential role in SBA [14,22,23]. Especially, a lack of nonsense mutations has been noted. In our data, *APC* was relatively frequently mutated (27/106, 25.5%), as reported in recent studies [7,8]. Furthermore, the majority of *APC* mutations in our data were protein-truncating, and the mutation frequency varied between the small bowel segments. We also detected 21 deletions overlapping the *APC* locus solely in MSS tumors, three of which co-occurred with a truncating mutation. Although the overall mutation rate was lower than in CRC, our results support the importance of *APC* also in the pathogenesis of SBA. Additionally, *APC* has been reported to be less frequently mutated in MSI than in MSS CRC [17], whereas in our set *APC* was more frequently mutated in MSI SBAs. Recently, *APC* mutations were reported to occur exclusively in SBA patients without IBD [7]. Our results indicate, however, that a subset of SBA patients with IBD have inactivating *APC* mutations.

Among the most significantly mutated genes was also *BRAF*, a well-known oncogene mutated in various cancers, such as melanoma (44%), CRC (10%), and lung adenocarcinoma (10%) [24]. In our study, *BRAF* was mutated in 11 tumors (11/106, 10.4%), which is consistent with current literature [6–8,22]. Instead of the most common activating mutation, V600E, we identified two atypical mutation hotspots, G469A and D594A/G/N, the first having been shown to activate and the latter to inhibit BRAF kinase activity [25,26]. These hotspot mutations were present exclusively in MSS tumors, as indicated previously [27]. In addition, the observed surrounding mutations were also either activating (K601N) or inactivating (G466E & G596R). Like activating *BRAF* mutations, the inactivating mutations are also thought to activate the MEK/ERK pathway, albeit through activation of the related family member CRAF [28]. Heidorn *et al*. showed that the kinase-dead BRAF needs activated RAS to induce BRAF binding to CRAF [26]. This could explain the co-occurrence of mutant *KRAS* with the kinase-silencing and truncating *BRAF* mutations. Co-occurrence of kinase-impaired *BRAF* with mutant *KRAS* has been reported in various malignancies [25–27].

A recent study on SBA reported only 10.3% of *BRAF* mutations to be V600E, whereas we identified none, together highlighting the importance of atypical *BRAF* mutations in SBA [7]. Of note, metastatic CRCs harboring non-V600 *BRAF* mutations have been shown to display distinct clinicopathologic features and an improved overall survival compared to V600E mutated CRCs [29]. These non-V600 mutations are also common e.g. in lung adenocarcinomas and melanomas [25]. Investigation is undergoing to elucidate how different non-V600 *BRAF* mutants respond to therapy. These tumors are unlikely to respond to selective BRAF inhibitors but might respond to MEK or pan-RAF inhibitors [26,30]. Our results suggest that screening for atypical *BRAF* mutations may be clinically relevant, since they can be at least as frequent as *BRAF* V600E and help guide personalized treatment choices.

Exome data analysis also revealed other significantly mutated genes previously linked to SBA (e.g. *SMAD4)*, recently reported potential driver genes (e.g. *SOX9*, *ATM*, and *ARID2*), and novel candidates (e.g. *ACVR2A*, *ACVR1B*, *BRCA2*, and *SMARCA4)* that have not previously been linked to SBA [5,7]. For example *ATM*, one of the recently reported potential SBA driver genes, was ranked the 10th most significant gene in our MSS tumor set, with half of the mutations being truncating. *ATM* is also significantly mutated in lung adenocarcinomas, kidney clear cell carcinomas, and prostate adenocarcinomas [7,31]. *ATM* has been implicated as a barrier to dysplastic growth in bowel tumors [32]. It has also potential clinical relevance as a biomarker to predict PARP inhibitor sensitivity.

The novel candidate SBA driver genes, *ACVR2A*, *ACVR1B*, *BRCA2*, and *SMARCA4*, have been previously implicated as drivers in various other human malignancies. *ACVR2A*, a known MSI target gene, encodes for a type II activin receptor that is involved in activin-mediated signalling [33]. Indeed, *ACVR2A* was the most frequently mutated known cancer gene in our MSI SBAs. *ACVR2A* was also among the significantly mutated genes in MSS tumors with mutations affecting the TGF-β receptor and the protein kinase domains. *ACVR2A* forms an activin receptor complex with *ACVR1B*. *ACVR1B* encodes for a type I activin receptor that regulates many biological processes, including extracellular matrix production and cell growth inhibition [34]. All the observed *ACVR1B* mutations, except one in TGF-β receptor GS domain, hit the protein kinase domain. *ACVR1B* has been shown to be significantly mutated in CRC, for instance [31]. It has also been indicated *in vivo* as a tumor suppressor in pancreatic cancer [35]. Our results implicate both *ACVR2A* and *ACVR1B* as candidate therapeutic targets in SBA.

*BRCA2* encodes for a known tumor suppressor that is involved in the repair of double-strand breaks in DNA by homologous recombination [36]. Over a thousand mutations have been found throughout this gene. Inactivating germline mutations in this gene are associated with the hereditary breast-ovarian cancer syndrome [37]. Somatic *BRCA2* mutations have been found e.g. in melanoma, where these mutations have been found to correlate with anti-PD-1 responsiveness [38]. The mutations observed here, the majority of which protein-truncating, were scattered along the gene. The gene has also further clinical relevance since drugs targeting *BRCA1* and *BRCA2* mutations are being developed [39].

*SMARCA4* encodes for one of the main catalytic subunits of mammalian SWI/SNF chromatin remodelling complex [40]. Here, most *SMARCA4* mutations located in known gene domains with a mutation hotspot in helicase C-domain. *SMARCA4* has been suggested to be a tumor suppressor, but some studies have reported *SMARCA4* overexpression in advanced cancers, proposing *SMARCA4* to be pro-oncogenic [41]. Additionally, the loss of *SMARCA4* seems to attenuate aberrant Wnt signalling in *APC*-deficient small bowel epithelium in mice [42]. *SMARCA4* has been shown to be significantly mutated in lung adenocarcinomas and esophageal cancer [31]. Chromatin regulators, in general, have been suggested as biomarkers for drug response and therapeutic targets [43].

*ERBB2* was mutated in altogether 14% of the tumors (15/106). We identified four known mutation hotspots (S310F/Y, R678Q, L755S, and V842I), of which R678Q has not been previously shown to be mutated in SBA [5–7]. These mutation hotspots have also been detected in other cancer types, such as breast and bladder cancer [16]. Of these, S310F, L755S and V842I are associated with drug sensitivity [44]. One of R678Q mutations co-occurred with another *ERBB2* hotspot mutation in our set. This phenomenon has been reported previously, suggesting that in these cases R678Q might provide additional selective value [44]. In addition to activating point mutations, oncogenic activation of *ERBB2* can occur through amplification and overexpression. We detected localized and strong amplification of *ERBB2* in four samples, two of which co-occurred with a hotspot mutation. Consequently, the prevalence of *ERBB2* alterations in SBAs is likely to be even higher.

The other members of the ERBB family are also commonly overexpressed, amplified, or mutated in various cancers [45]. We detected hotspot mutations in *ERBB3* (V104M/L and S846I) and *ERBB4* (L798R/P), albeit with lower frequency than in *ERBB2*. These hotspots have been previously reported in e.g. gastric adenocarcinomas (GA) and CRC but, to our knowledge, not in SBA [46]. Of these, V104M/L has been shown to be a statistically significant mutation hotspot and, along with S846I, to promote oncogenic signalling [16,46]. Many approved therapies targeting ERBB2 and EGFR receptors are in clinical use [45]. Multiple ERBB family members have potential clinical relevance, as therapies targeting them are currently being developed [46,47]. Particularly, *ERBB2* can be considered as a potential therapeutic target in SBA.

In addition to identifying possible single therapeutic target genes, we examined the essential pathways in SBA. Of the well-known cancer related pathways, PI3K/AKT and ERBB signalling were affected in most of MSS tumors. Comparison between the small bowel segments uncovered shared mutated pathways although there was some variability in the order of mutation frequencies. For example, in our set of duodenal SBAs, ERBB signalling was the most frequently affected pathway, followed by ERK/MAPK signalling. In jejunal and ileal tumors the most frequently mutated pathway was PI3K/AKT signalling, followed by ERK/MAPK signalling in jejunal and Wnt/β-catenin signalling in ileal tumors. Though these results may reflect variation between the tumor subgroups, more work is still needed to robustly elucidate the differences.

We performed, to our knowledge, the first comprehensive signature analysis of SBA and identified four mutational signatures: 1A, 6, 17, and U2. Signature 1A is proposed to be a result of spontaneous deamination of 5-methylcytosine, whereas the process causing signature 17 is still unknown [19]. The observed association of signature 1A with older age at diagnosis has been reported in other tumor types, such as medulloblastoma and gastric cancer [19]. We also observed a previously unreported association between increased signature 1A exposure and jejunal tumor location, even though patients with jejunal tumors were, on average, younger than those with duodenal and ileal tumors. This may suggest regional differences in DNA methylation or in the rate of cell division between different segments. Signature U2 has been reported in liver, prostate, and kidney chromophobe cancers, but thus far has been unvalidated. However, we were able to inspect read sequences in our dataset and thus validate mutations in this signature class. These results revealed that SBA, CRC, and gastric cancer share features in their signature content. Signatures 1A and 17 have been reported in both CRC and gastric cancer studies [19,48]. Signature-wise SBA seems to closely resemble CRC, since the majority of associated signatures overlap. Although the small and large bowel represent different environments, they may share comparable exposures that could explain similarities in the tumors’ signature content. Many additional signatures have been associated with gastric cancer, and thus signature-wise they differ from SBAs.

Compared to GAs and CRCs, SBAs displayed similar mutation frequencies of certain driver genes, such as *TP53*, *SMAD4*, and *PIK3CA* [17,18]. Additionally, the proportions of MSI tumors were similar in these tumor types. Thus, MSI testing should be also considered in SBA in view of benefit from immunotherapy [49]. We also found that, as in CRC, patients with MSI tumors had a longer disease-specific survival than patients with MSS SBA [50]. On the contrary, many notable differences between SBA and GA/CRC were observed. For instance, the frequency of *KRAS* mutants resembled that of CRC, but was clearly higher than that in GAs. The *APC* mutation frequency differed between the three malignancies, and seemed to increase along the GI-tract, confirming previous results [7]. Also, the *BRAF* mutation spectrum varied markedly, since SBA was the only one where *BRAF* mutations consisted mainly of atypical mutations. Our results support the notion that SBA is a distinct entity with a unique set of significantly mutated genes. Despite our large population-based dataset, no obvious genetic reason for the low incidence of SBA compared to CRC was detected.

The Finnish Cancer Registry allowed us to collect information on all SBA cases in Finland. Due to insufficient tumor material or low tumor percentage in some cases, we were unable to include every patient diagnosed during the selected years. However, we believe that the sample material is approximately representative of the population. Duodenum has been reported to be the most common location of SBAs. Duodenal tumors were slightly underrepresented due to: 1) exclusion of tumors from the papillary region (which are classically grouped together with duodenal tumors), and 2) the fact that some duodenal tumors were only biopsied and had too little material for exome sequencing. Besides this, all segments were fairly well-represented. Due to the lack of corresponding normal samples, we used strict filtering methods for somatic variant calling. However, we recognize that the data may contain some rare germline variants. Despite exome sequencing being highly informative, we acknowledge that non-coding genetic driver mechanisms remain currently unaddressed.

This large population-based study elucidated the molecular basis of SBA through exome sequencing. The results singled out many potential therapeutic targets that could be exploited when developing treatments for SBAs. These include both currently targetable genes (*BRAF*, *ERBB2*, and *BRCA2*) and novel candidates including *ERBB3*, *ERBB4*, *PIK3CA*, *KRAS*, *ATM*, *ACVR2A*, *ACVR1B*, and *SMARCA4*. In addition to *KRAS*, we detected multiple genes that may predict resistance to anti-EGFR therapy, such as *BRAF*, *ERBB2*, and *PIK3CA*. Additionally, this was the first large-scale pursuit to compare the primary tumors from all three small bowel segments. Although the tumors shared somewhat similar characteristics, differences were noted. The results presented here provide further evidence that SBA is a genetically distinct tumor entity. Observed heterogeneity in the mutational landscape indicates that several driver genes play a role in the biology of SBA. These results take forward our understanding of the pathogenesis of SBA and ultimately should be useful for the management of the disease.

## Materials and methods

### Ethics statement

The study has been reviewed and approved by the Ethics Committee of the Hospital District of Helsinki and Uusimaa, Finland (408/13/03/03/2009). Authorisation from the National Supervisory for Welfare and Health was obtained for genetic studies on the samples, as determined in the National legislation. This study has been conducted according to the Declaration of Helsinki.

### Patient cohort

We compiled from the Finnish Cancer Registry information on all patients diagnosed with SBA in Finland during years 2003–2011. This registry maintains a nationwide database on all cancer cases diagnosed since 1953, and has almost complete coverage [51]. In order to focus solely on small bowel tumors, we excluded tumors of the papillary region (n = 31) since they might have originated in the pancreas or the biliary tract. Cases reported only by autopsy (n = 20) and cases without histopathological confirmation of small bowel primary tumor (n = 25) were also excluded from the study, and 162 cases remained. From these we selected all cases with available tumor material and tumor content of at least 50%. In total, 55 SBA cases were excluded due to these reasons and one due to low sequencing depth. The final set consisted of 106 out of 162 (65%) confirmed SBA cases (excluding autopsies). All relevant medical records, including follow-up information for survival analysis, were available for all the cases.

### DNA extraction

Hematoxylin-eosin staining was performed to estimate tumor percentages. To reach maximal tumor percentage, macrodissection was conducted, when possible, to remove non-malignant tissue. Genomic DNA extractions from formalin-fixed and paraffin-embedded (FFPE) blocks were performed using either a standard phenol-chloroform isolation method or GeneRead FFPE-kit according to manufacturer’s instructions (QIAGEN, Hilden, Germany). DNA concentration was determined with Qubit double-stranded DNA BR Assay Kit (Thermo Fisher Scientific, Waltham, MA, USA) and purity with NanoDrop8000 (Thermo Fisher Scientific).

### Exome capture and sequencing

Exome libraries were prepared with KAPA Hyper Prep Kit (Kapa Biosystems, Wilmington, MA, USA). Coding exons and untranslated regions (UTRs) of the genome (94 megabases) were enriched with NimbleGen SeqCap EZ Exome Library v3 Kit (Roche NimbleGen, Madison, WI). Paired-end sequencing with read lengths of 75 base pairs with a median depth of 40x (range, 33x to 62x) was performed with Illumina HiSeq 4000 (Illumina Inc., San Diego, CA) in Karolinska Institutet, Sweden. At least 85% of the exome target was covered by a minimum of 10 reads in all except two samples (SIA137, 82%; SIA196, 83%).

### Read mapping and variant calling

The quality of raw sequencing data was examined with FastQC v.0.10.0 (http://www.bioinformatics.babraham.ac.uk/projects/fastqc/) and QualiMap v.2.1 (http://qualimap.bioinfo.cipf.es/) [52]. Trim Galore! v.0.3.07 (http://www.bioinformatics.babraham.ac.uk/projects/trim_galore/) was used to remove the 3’ ends of reads with high adapter similarity. The trimmed reads were then mapped to the integrated 1000 Genomes Phase 2 GRCh37/hg19 reference assembly with Burrows-Wheeler Aligner (BWA)–MEM v.0.7.12 (http://bio-bwa.sourceforge.net/) [53]. BamUtil v.1.0.13 (http://genome.sph.umich.edu/wiki/BamUtil#Releases) ClipOverlap was used to clip overlapping read pairs. Duplicate reads were removed using Samtools version 1.0 (http://www.htslib.org/) rmdup on both paired-end and single-end reads [54]. Aligned reads were locally realigned with the Genome Analysis ToolKit (GATK) v.3.5 (https://www.broadinstitute.org/gatk/) IndelRealigner [55]. GATK BaseRecalibrator was utilized to recalculate base scores. After realignment the final indel and single nucleotide variant (SNV) calls were produced with the GATK HaplotypeCaller using a Phred-scaled confidence threshold (stand_call_conf) of 1.0.

### Somatic variant analysis

Since our exome data consisted of only tumors, we utilized methods similar to those in Hiltemann *et al*. for discriminating somatic variants; the approach removed >96% of the germline variants when corresponding normals were not available [56]. However, additional filtering steps as well as larger population and sequencing pipeline specific datasets were used in this study. Putative somatic variants were extracted by filtering SNV and indel calls against whole-genome and exome samples of the GnomAD dataset (n = 138,632) [57]. First, we excluded all variants found in Finnish whole-genome samples (n = 1,747). This set was utilized separately to remove common as well as population-specific variation equally at the whole targeted region, as the exome data did not fully cover our targets. Then we applied the full GnomAD dataset (exomes and genomes) using allele frequency threshold; variants with allele frequency more than 0.0001 were excluded. For SNVs, matching chromosomal position and base change were required for exclusion. Indels were excluded in cases of overlapping occurrences. Additional filtering was performed with 183 in-house whole-genome sequencing samples (normal solid tissue or peripheral blood) to remove sequencing platform and variant calling pipeline specific errors. We refined remaining variant calls against a pooled set of whole-genome sequencing data (median ~40x coverage/sample) from 10 blood samples by excluding any SNV call which was found in three or more reads in the pooled data. Indel calls were filtered out if two or more samples had more than three reads calling an indel at 100 base pairs (read length) from the indel locus. This step was done to exclude low allelic fraction artefacts in regions prone to sequencing errors. Only variants within the targeted region of NimbleGen SeqCap EZ Exome Library v3 Kit were analyzed.

BasePlayer [58] was utilized to visualize and analyze the data (allele frequency and quality filtering, allelic imbalance, gene annotation, and calculation of variant statistics). Variant filtering parameters are listed in S8 Table. Ensembl version 87 (GRCh37) was used for gene annotation. Mutation calls have been deposited in the EGA database (EGAS00001002559).

### OncodriveFML

We used OncodriveFML v.2.0.2 [59] to perform significance analysis for somatic mutations within the coding DNA sequence (CDS). OncodriveFML is a permutation-based method that compares a region’s mean functional impact score to its null distribution by randomizing observed mutations. Protein-coding CDS regions were obtained from Gencode release 19 (http://www.gencodegenes.org/). The resulting regions were then merged using bedtools (v.2.25.0). The method's default scoring framework, CADD [60], was used. OncodriveFML’s default configurations were applied, with the genomic elements file defined as “coding” and the sequencing type defined as “whole exome sequencing”. The focus was, solely, on genes mutated in at least four tumors. Quantile-quantile plots are presented in S7 Fig. Inflation factors for *P*-value distributions were estimated using the R package GenABEL v.1.8–0. The Benjamini-Hochberg method was applied to adjust for false discovery rate (FDR).

### Sanger sequencing

All non-synonymous mutations in the novel candidate genes (*ACVR2A*, *ACVR1B*, *BRCA2*, and *SMARCA4*) used in OncodriveFML analysis and genes with a clear mutation hotspot pattern (*ERBB2* and *BRAF*) were selected for validation with Sanger sequencing. Primers were designed using Primer3Plus [61]. Each PCR reaction was performed in triplicates to ensure consistency of the observations. Sequencing reactions were carried out with the Big Dye Terminator v.3.1 kit (Applied Biosystems, Foster City, CA, USA) on an ABI3730 Automatic DNA Sequencer (FIMM Technology Center and DNA sequencing and Genomics laboratory, Institute of Biotechnology, Helsinki, Finland). The sequence graphs were analyzed both with the Mutation Surveyor–software (version v4.0.8, Softgenetics, State College, PA) and manually.

Validation was successfully performed for altogether 49/54 mutations. From two tumors (SIA137 and SIA98) no DNA material was left for validation. For 47/49 mutations, we had just enough DNA material from the corresponding normal samples to validate their somatic status. All except two mutations in *BRCA2* were validated as somatic. These two rare germline variants (ExAC MAF = 0.00002 & 0.00005) were excluded from the whole study. Even after the removal of these two mutations, *BRCA2* remained in the top 25 genes in the OncodriveFML re-run.

### Allelic imbalance analysis

AI regions were called using germline SNVs of the whole sample set of 106 SIA tumors. We selected SNVs for the analysis based on following criteria:

- rs-coded
- 10 or more coverage at variant call locus
- not defined as somatic in this study
- within exome target regions
- does not overlap with regions prone to false allelic imbalance calls (see control analysis below)

B allele frequency segmentation (BAFsegmentation) algorithm (described in Staaf *et al*. [62]) was utilized to call AI regions with parameters: non_informative = 0.97, ai_threshold = 0.6, ai_size = 4, triplet_threshold = 0.8. BAF value was calculated from allelic depth fields of VCF file (ALT calls / total coverage).

First, we performed control analysis with 80 normal exomes using the same parameters to detect possible technical artefacts caused by low-complexity genomic loci and usage of exome variant data, which has limited power to detect AI. Control analysis revealed genomic regions more prone to false calls (e.g. centromeres and chromosome ends). Variants overlapping these regions were excluded from the tumor analysis. In addition, we observed median coverage differences between chromosomes (e.g. median coverages across all samples in chromosome 1 and 16 was 38 and 30, respectively). This information was used for chromosome-specific coverage normalization in calculation of log-R ratios for tumor variants. Median coverage for X chromosome was calculated by using only female samples.

We ran BAFsegmentation for tumor samples twice. The first run was performed to detect AI regions to get as accurate median coverage for all samples as possible. Median coverages were calculated using all variants, which did not overlap with called AI regions. Variant-specific log-R ratios were calculated using following formula (1):

log2(varCoverage/(sampleMedian*chromNormalize[chr])) (1)

*varCoverage* was obtained from coverage field (DP) in VCF-file. *sampleMedian* is sample-specific median coverage value of all chromosomes (AI regions excluded). *chromNormalize*[*chr*] corresponds to chromosome specific coverage normalization coefficient, which was processed in control analysis. Second, and last BAFsegmentation run was performed using refined log-R ratios. AI events with median log-R ratios higher than 0.1 were considered as gains and events equal or less than 0.1 were considered as losses (including copy number neutral loss of heterozygosity).

### Mutation signature analysis

First, we performed signature analysis on 106 SBAs, as in Katainen *et al*., using non-negative matrix factorization of six substitution types in 5′-Xp(C/T)pY-3′ for any nucleotides X and Y [48,63]. All variants within exome target regions were used, including UTRs. We computed the exposure of extracted signatures for each 106 SBAs as a projection of the mutation matrix to the signature weight matrix. The obtained signatures (*p)* were compared to the published signatures (*q)* of Alexandrov *et al*. by mean Kullback-Leibler divergence (*D*_KL_(*p*||*q*)+*D*_KL_(*q*||*p*)]/2. Fifteen samples displayed the MSI signature (Signature 6), consistent with the division of tumors based on the exome data (S2 Table). Mutation signature analysis was subsequently performed in 91 MSS SBAs.

### Ingenuity Pathway Analysis

Ingenuity Pathway Analysis (IPA) version 39480507 was used to determine the frequency of known cancer pathways affected in the MSS tumors. IPA was utilized to define genes linked to each pathway. All genes with at least one non-synonymous mutation were included in the analysis.

### Statistical analysis of clinical data

We used R v.3.4.1 to analyze clinical variables. Fisher’s exact test was used to test for independence of categorical variables. Differences in continuous variables were assessed with the Mann-Whitney U test. Disease-specific survival was analyzed by Cox proportional hazards regression with Firth's penalized likelihood (coxphf package v.1.12). Per-tumor mutation counts attributable to mutational signatures were estimated in MSS tumors, and their associations with clinical features were modeled using negative binomial regression (MASS package v.7.3–47). All *P*-values are two-sided and unadjusted for multiple comparisons. *P*-value <0.05 was regarded as statistically significant.

## Supporting information

S1 Table Cox proportional hazards model for disease-specific survival (a) and negative binomial models for allelic imbalance and mutational signatures (b-c).
(PDF)

S2 Table Mutation statistics from exome sequencing data. This table includes sample-wise statistics for all somatic variants within the targeted region.
(XLSX)

S3 Table OncodriveFML results of the MSS tumors (a) and mutation content of the genes that received *P* < 0.05 in the OncodriveFML analysis (b).
(XLSX)

S4 Table Comparison of clinicopathological characteristics between (a) BRAF and non-BRAF mutants and (b) ERBB2 and non-ERBB2 mutants.
(PDF)

S5 Table Signature analysis: (a) division of MSI and MSS tumors, (b) the MSS exposures, and (c) the signature weights (MSS), and (d) the signature weights (MSI).
(XLSX)

S6 Table Pathway analysis: (a) Frequencies of mutated pathways in the tumor set, (b) List of genes (with at least one mutation in MSS tumors) per pathway.
(PDF)

S7 Table Comparison between the three small bowel segments. (PDF)

S8 Table Filtering criteria for SNVs and indels. Recommended GATK hard filters.
(PDF)

S1 Fig **Kaplan-Meier estimates of disease-specific survival according to a) MMR status, b) age at operation, c) stage, and d) sex.** Eighteen patients were omitted due to missing data, (n = 88). The total duration of follow-up was 379 person-years, and 53 deaths from SBA were observed. In Cox regression model with adjustment for sex, tumor stage, and age at operation, MSI tumors were associated with better disease-specific survival compared to MSS tumors (hazard ratio (HR), 0.111; 95% CI, 0.0292–0.419; *P* = 1.20x10^-3^). *P*-values for unadjusted log-rank tests are shown in the figure.
(PDF)

S2 Fig Somatic mutation prevalence. The mutation burden in the whole set, n = 106. Median value (red line) = 3.96.
(PDF)

S3 Fig AI events in 91 MSS SBAs. Genes highlighted in our study (the 25 highest-ranking genes in OncodriveFML and the ERBB-family genes) (red) and cancer census genes near visible AI peaks (purple) are depicted in the graphs.
(PDF)

S4 Fig Landscape of mutations and AI events in MSS SBAs. The figure includes the 25 highest-ranking genes in OncodriveFML and the ERBB-family genes. Different colors distinguish between the different types of AI events. Non-synonymous mutations are marked with a black dot.
(PDF)

S5 Fig AI events in the *ERBB2* and chromosome 17. Four tumors showed a strong localized amplification in *ERBB2*, of which two tumors harbored also *ERBB2* mutation (SIA82, V842I and SIA137, S310Y).
(PDF)

S6 Fig Comparison of signature 1A and age at diagnosis between tumors from different segments. Exposure to signature 1A was highest in jejunal tumors even though the median age at diagnosis was lower in patients with jejunal tumor compared to patients with duodenal or ileal tumors.
(PDF)

S7 Fig Quantile-quantile plots for MSS (n = 91) OncodriveFML analysis, (a) with all the genes included in the initial run and (b) after filtering the data to contain genes mutated in at least four samples.
(PDF)
