# Supplementary material for: Exome-wide somatic mutation characterization of small bowel adenocarcinoma
Source: PLoS Genet. 2018 Mar 9;14(3):e1007200. doi: 10.1371/journal.pgen.1007200 (PMC5871010; doi:10.1371/journal.pgen.1007200)
Supplement: S1 Table — Cox proportional hazards model for disease-specific survival (a) and negative binomial models for allelic imbalance and mutational signatures (b-c). (PDF) [file pgen.1007200.s001.pdf]

S1 Table. Cox proportional hazards model for disease-specific survival (a) and negative binomial models for allelic imbalance and mutational signatures (b-c).

Table a. Cox proportional hazards model for disease-specific survival. Firth's penalized likelihood was applied. MSI, microsatellite instability.

Model summary: N = 88, 53 events, 18 observations deleted due to missing data

		95% confidence bounds					
	Hazard ratio	Lower	Upper	P-value			
MSI[a]	0.111	0.0292	0.419	1.20e-03	**	Likelihood ratio test	81.5 on 7 degrees of freedom
Stage II[b]	1.68	0.271	10.4	0.0577		Wald test	58.6 on 7 degrees of freedom
Stage III[b]	2.53	0.404	15.8	0.322			
Stage IV[b]	36.3	5.65	233	1.53e-04	***	Significance codes: 0 '***' 0.001 '**' 0.01 '*' 0.05 '.' 0.1 ' ' 1	
Age at operation 60-70 years	2.38	1.09	5.18	0.0292	*	coxphf package v. 1.12	
Age at operation >70 years	6.00	2.73	13.2	8.14e-06	***		
Male	1.68	0.951	2.98	0.0741	.		

a) Reference level: microsatellite-stable.

b) Reference level: stage I.

Table b. Negative binomial model for the number of allelic imbalance events.

Model summary: N = 106, 10 observations deleted due to missing data

		95% confidence bounds					
	exp(Coefficient)	Lower	Upper	P-value			
Intercept	20.3	8.48	48.8	1.45e-11	***	Null deviance	136 on 95 degrees of freedom
Age at operation (/10 years)[a]	1.05	0.934	1.18	0.411		2 x log-likelihood	-754
Female	0.890	0.679	1.17	0.399		AIC	768
MSI[b]	0.251	0.160	0.394	1.95e-09	***	Theta estimate	2.51
Jejunal location[c]	0.971	0.691	1.36	0.864			
Ileal location[c]	0.872	0.578	1.32	0.514		Significance codes: 0 '***' 0.001 '**' 0.01 '*' 0.05 '.' 0.1 ' ' 1	

MASS package v. 7.3-47

a) Age was modeled as a continuous log-linear predictor.

b) Reference level: microsatellite-stable.

c) Reference level: duodenum.

Table c. Negative binomial models for mutational signatures.

Model summaries: N = 91, 7 observations deleted due to missing data

Signature 1A

		95% confidence bounds					
	exp(Coefficient)	Lower	Upper	P-value			
Intercept	25.2	13.0	49.1	1.77e-21	***	Null deviance	119 on 83 degrees of freedom
Age at operation (/10 years)[a]	1.20	1.10	1.32	4.32e-05	***	Residual deviance	87.0 on 79 degrees of freedom
Female	1.26	1.03	1.54	0.0259	*	2 x log-likelihood	-907
Jejunal location[b]	1.66	1.29	2.14	7.17e-05	***	AIC	919
Ileal location[b]	1.16	0.852	1.58	0.348		Theta estimate	4.80

Signature 17

			95% confidence bounds					
		exp(Coefficient)	Lower	Upper	P-value			
With SIA80	Intercept	28.0	4.13	190	6.46e-04	**	Null deviance	113 on 83 degrees of freedom
	Age at operation (/10 years)[a]	0.897	0.694	1.16	0.408		Residual deviance	99.6 on 79 degrees of freedom
	Female	0.994	0.557	1.77	0.984		2 x log-likelihood	-684
	Jejunal location[b]	2.75	1.33	5.67	6.37e-03	**	AIC	696
	Ileal location[b]	0.960	0.394	2.34	0.928		Theta estimate	0.570

			95% confidence bounds					
		exp(Coefficient)	Lower	Upper	P-value			
Without SIA80	Intercept	15.4	2.82	84.4	1.59e-03	***	Null deviance	105 on 82 degrees of freedom
	Age at operation (/10 years)[a]	0.950	0.757	1.19	0.662		Residual deviance	95.8 on 78 degrees of freedom
	Female	1.77	1.05	2.96	0.0308	*	2 x log-likelihood	-642
	Jejunal location[b]	1.54	0.805	2.93	0.192		AIC	654
	Ileal location[b]	0.858	0.389	1.89	0.705		Theta estimate	0.735

Signature U2

		95% confidence bounds					
	exp(Coefficient)	Lower	Upper	P-value			
Intercept	92.0	24.2	350	3.28e-11	***	Null deviance	100 on 83 degrees of freedom
Age at operation (/10 y)[a]	1.00	0.838	1.20	0.976		Residual deviance	96.7 on 79 degrees of freedom
Female	0.969	0.647	1.45	0.880		2 x log-likelihood	-879
Jejunal location[b]	0.657	0.396	1.09	0.103		AIC	891
Ileal location[b]	0.688	0.371	1.27	0.234		Theta estimate	1.16

Significance codes: 0 '***' 0.001 '**' 0.01 '*' 0.05 '.' 0.1 ' ' 1
MASS package v. 7.3-47

a) Age was modeled as a continuous log-linear predictor.

b) Reference level: duodenum.
