# Supplementary material for: Exome-wide somatic mutation characterization of small bowel adenocarcinoma
Source: PLoS Genet. 2018 Mar 9;14(3):e1007200. doi: 10.1371/journal.pgen.1007200 (PMC5871010; doi:10.1371/journal.pgen.1007200)
Supplement: S4 Table — Comparison of clinicopathological characteristics between (a) BRAF and non-BRAF mutants and (b) ERBB2 and non-ERBB2 mutants. (PDF) [file pgen.1007200.s004.pdf]

**S4 Table. Comparison of clinicopathological characteristics between (a) *BRAF* and non-*BRAF* mutants and (b) *ERBB2* and non-*ERBB2* mutants.**

**Table a.** Comparison of clinicopathological characteristics between *BRAF* and non-*BRAF* mutants. The table includes all samples (n=106).

| **Characteristics** | | ***BRAF* wild-type (n=95)** | | ***BRAF* mutated (n=11)** | | ***P*** |
|---|---|---|---|---|---|---|
| | | **n** | **%** | **n** | **%** | |
| **Sex** | | | | | | |
| | Female | 46 | 48.4 | 4 | 36.4 | 0.534 |
| | Male | 49 | 51.6 | 7 | 63.6 | |
| **Age (years)** | | | | | | |
| | Median | 63 | | 60 | | 0.143 |
| | Range | 24-86 | | 26-74 | | |
| **MMR status** | | | | | | |
| | MSS | 81 | 85.3 | 10 | 90.9 | 1 |
| | MSI | 14 | 14.7 | 1 | 9.1 | |
| **Location** | | | | | | |
| | Duodenum | 25 | 26.3 | 1 | 9.1 | 0.458 |
| | Jejunum | 45 | 47.4 | 7 | 63.6 | |
| | Ileum | 17 | 17.9 | 1 | 9.1 | |
| | NA | 8 | 8.4 | 2 | 18.2 | |
| **Grade** | | | | | | |
| | 1 | 16 | 16.8 | 2 | 18.2 | 0.822 |
| | 2 | 54 | 56.8 | 6 | 54.5 | |
| | 3 | 17 | 17.9 | 3 | 27.3 | |
| | NA | 8 | 8.4 | 0 | 0.0 | |
| **T** | | | | | | |
| | 1-2 | 8 | 8.4 | 0 | 0.0 | 0.591 |
| | 3-4 | 77 | 81.1 | 11 | 100 | |
| | NA | 10 | 10.5 | 0 | 0.0 | |
| **N** | | | | | | |
| | 0 | 28 | 29.5 | 3 | 27.3 | 1 |
| | 1-2 | 42 | 44.2 | 5 | 45.4 | |
| | NA | 25 | 26.3 | 3 | 27.3 | |
| **M** | | | | | | |
| | 0 | 51 | 53.7 | 6 | 54.5 | 1 |
| | 1 | 36 | 37.9 | 5 | 45.5 | |
| | NA | 8 | 8.4 | 0 | 0.0 | |
| **Stage** | | | | | | |
| | 1 | 4 | 4.2 | 0 | 0.0 | 1 |
| | 2 | 19 | 20.0 | 3 | 27.3 | |
| | 3 | 22 | 23.2 | 3 | 27.3 | |
| | 4 | 36 | 37.9 | 5 | 45.4 | |
| | NA | 14 | 14.7 | 0 | 0.0 | |

**Table b.** Comparison of clinicopathological characteristics between *ERBB2* and non-*ERBB2* mutants. The table includes all samples (n=106).

| **Characteristics** | | ***ERBB2* wild-type (n=91)** | | ***ERBB2* mutated (n=15)** | | ***P*** |
|---|---|---|---|---|---|---|
| | | **n** | **%** | **n** | **%** | |
| **Sex** | | | | | | |
| | Female | 41 | 45.1 | 9 | 60.0 | 0.403 |
| | Male | 50 | 54.9 | 6 | 40.0 | |
| **Age (years)** | | | | | | |
| | Median | 62 | | 66 | | 0.0801 |
| | Range | 24-86 | | 49-86 | | |
| **MMR status** | | | | | | |
| | MSS | 81 | 89 | 10 | 66.7 | 0.0368 |
| | MSI | 10 | 11.0 | 5 | 33.3 | |
| **Location** | | | | | | |
| | Duodenum | 23 | 25.3 | 3 | 20.0 | 1 |
| | Jejunum | 45 | 49.5 | 7 | 46.7 | |
| | Ileum | 16 | 17.6 | 2 | 13.3 | |
| | NA | 7 | 7.7 | 3 | 20.0 | |
| **Grade** | | | | | | |
| | 1 | 15 | 16.5 | 3 | 20.0 | 0.432 |
| | 2 | 50 | 54.9 | 10 | 66.7 | |
| | 3 | 19 | 20.9 | 1 | 6.7 | |
| | NA | 7 | 7.7 | 1 | 6.7 | |
| **T** | | | | | | |
| | 1-2 | 8 | 8.8 | 0 | 0.0 | 0.599 |
| | 3-4 | 74 | 81.3 | 14 | 93.3 | |
| | NA | 9 | 9.9 | 1 | 6.7 | |
| **N** | | | | | | |
| | 0 | 26 | 28.6 | 5 | 33.3 | 1 |
| | 1-2 | 40 | 44.0 | 7 | 46.7 | |
| | NA | 25 | 27.4 | 3 | 20.0 | |
| **M** | | | | | | |
| | 0 | 49 | 53.8 | 8 | 53.3 | 1 |
| | 1 | 35 | 38.5 | 6 | 40.0 | |
| | NA | 7 | 7.7 | 1 | 6.7 | |
| **Stage** | | | | | | |
| | 1 | 4 | 4.4 | 0 | 0.0 | 0.965 |
| | 2 | 18 | 19.8 | 4 | 26.7 | |
| | 3 | 21 | 23.1 | 4 | 26.7 | |
| | 4 | 35 | 38.4 | 6 | 40.0 | |
| | NA | 13 | 14.3 | 1 | 6.6 | |
