# Supplementary material for: Exome-wide somatic mutation characterization of small bowel adenocarcinoma
Source: PLoS Genet. 2018 Mar 9;14(3):e1007200. doi: 10.1371/journal.pgen.1007200 (PMC5871010; doi:10.1371/journal.pgen.1007200)
Supplement: S6 Table — Pathway analysis: (a) Frequencies of mutated pathways in the tumor set, (b) List of genes (with at least one mutation in MSS tumors) per pathway. (PDF) [file pgen.1007200.s006.pdf]

S6 Table. Pathway analysis: (a) Frequencies of mutated pathways in the tumor set, (b) List of genes (with at least one mutation in MSS tumors) per pathway.

Table a. Frequencies of mutated pathways in the tumor set.

MSS tumors (n=91)	no. of tumors	%
PI3K/AKT	77	84.6
ERBB	73	80.2
ERK/MAPK	72	79.1
Wnt/β-catenin	70	76.9
TGF-β	66	72.5
p53	63	69.2

Duodenum (n=24)	no. of tumors	%
ERBB	20	83.3
ERK/MAPK	18	75.0
PI3K/AKT	17	70.8
Wnt/β-catenin	16	66.7
TGF-β	15	62.5
p53	13	54.2

Jejunum (n=44)	no. of tumors	%
PI3K/AKT	42	95.5
ERK/MAPK	40	90.9
ERBB	38	86.4
Wnt/β-catenin	38	86.4
TGF-β	36	81.8
p53	35	79.5

Ileum (n=16)	no. of tumors	%
PI3K/AKT	12	75.0
Wnt/β-catenin	11	68.8
p53	10	62.5
TGF-β	9	56.3
ERK/MAPK	9	56.3
ERBB	9	56.3

Table b. List of genes (with at least one mutation in MSS tumors) per pathway.

ERBB Signalling

PIK3CA
PRKCQ
NRAS
PIK3C2G
PLCG1
EGF
ERBB3
KRAS
KLB
PTPN11
MAPK3
FGFR4
ERBB4
PLCG2
PIK3CG
PIK3CD
ERBB2
MAP2K1
PRKCB
ATM
PRKCA

PI3K Signalling

TP53
PIK3CA
NRAS
KRAS
NFKB2
PTEN
HSP90AB1
MAPK3
PIK3CG
PPP2R2B
TSC2
PIK3CD
CDKN1B
CTNNB1
MAP2K1

TGFβ Signalling

ZFYVE9
SMAD2
TGFBR1
NRAS
SMAD3
CREBBP
BMPR2
KRAS
BMPR1B
ACVR1B
INHBA
TGFBR2
BMPR1A
MAPK3
SMAD4
BMP7
MAP2K1
ACVR2A

ERK/MAPK Signalling

PIK3CA
NRAS
CREBBP
PIK3C2G
PLCG1
KRAS
RAPGEF4
KLB
NFATC1
BRAF
DOCK1
PLA2G4D
ELF3
PTPN11
MAPK3
FGFR4
PLCG2
PIK3CG
PPP2R2B
PIK3CD
MAP2K1
PRKCB
PRKCA
ATM

p53 Signalling

TP53
HDAC9
PRKDC
PIK3CA
PIK3C2G
KLB
PTEN
PTPN11
PIK3CG
FGFR4
PIK3CD
ATR
CTNNB1
ATM

Wnt/β Signalling

FZD10
TGFBR1
SOX10
BMPR2
BCL9
TGFBR2
WNT7A
CTNNB1
WNT5B
SOX5
TP53
LRP5
PPARD
CREBBP
DVL1
SOX11
APC
ACVR1B
CDH2
WNT8A
CDH12
PPP2R2B
SOX9
LEF1
LRP1
ACVR2A
