# Supplementary material for: Exome-wide somatic mutation characterization of small bowel adenocarcinoma
Source: PLoS Genet. 2018 Mar 9;14(3):e1007200. doi: 10.1371/journal.pgen.1007200 (PMC5871010; doi:10.1371/journal.pgen.1007200)
Supplement: S7 Table — (PDF) [file pgen.1007200.s007.pdf]

S7 Table. Comparison between the three small bowel segments.

MSS	Duodenum	Jejunum	Ileum	*P* -value
	n=24	n=44	n=16	
Celiac disease	1 (4.2%)	3 (6.8%)	0 (0.0%)	0.812
Crohn's disease	0 (0.0%)	2 (4.5%)	3 (18.8%)	0.0562
KRAS	9 (37.5%)	24 (54.5%)	5 (31.3%)	0.193
TP53	7 (29.2%)	25 (56.8%)	9 (56.3%)	0.0787
APC	9 (37.5%)	6 (13.6%)	5 (31.3%)	0.0528
ERBB2	3 (12.5%)	5 (11.4%)	1 (6.3%)	0.902
BRAF	1 (4.2%)	7 (15.9%)	1 (6.3%)	0.371

MSI	Duodenum	Jejunum	Ileum	*P* -value
	n=2	n=8	n=2	
Celiac disease	1 (50.0%)	4 (50.0%)	0 (0.0%)	0.717
Crohn's disease	0 (0.0%)	0 (0.0%)	0 (0.0%)	NA
KRAS	2 (100%)	3 (37.5%)	1 (50.0%)	0.697
TP53	1 (50.0%)	3 (37.5%)	0 (0.0%)	1
APC	1 (50.0%)	3 (37.5%)	1 (50.0%)	1
ERBB2	0 (0.0%)	2 (25.0%)	1 (50.0%)	1
BRAF	0 (0.0%)	0 (0.0%)	0 (0.0%)	NA
