# Supplementary material for: Exome-wide somatic mutation characterization of small bowel adenocarcinoma
Source: PLoS Genet. 2018 Mar 9;14(3):e1007200. doi: 10.1371/journal.pgen.1007200 (PMC5871010; doi:10.1371/journal.pgen.1007200)
Supplement: S8 Table — Recommended GATK hard filters. (PDF) [file pgen.1007200.s008.pdf]

S8 Table. Filtering criteria for SNVs and indels. Recommended GATK hard filters.

QualByDepth (QD)	< 2.0
FisherStrand (FS)	> 60.0
RMSMappingQuality (MQ)	< 40.0
MappingQualityRankSumTest (MQRankSum)	< -12.5
ReadPosRankSumTest (ReadPosRankSum)	< -8.0
Coverage	< 10
Allelic fraction (calls/coverage)	< 10%
Phred-scaled quality score (HaplotypeCaller)	< 20
