# Supplementary material for: Exome-wide somatic mutation characterization of small bowel adenocarcinoma
Source: PLoS Genet. 2018 Mar 9;14(3):e1007200. doi: 10.1371/journal.pgen.1007200 (PMC5871010; doi:10.1371/journal.pgen.1007200)
Supplement: S1 Fig — Kaplan-Meier estimates of disease-specific survival according to a) MMR status, b) age at operation, c) stage, and d) sex. Eighteen patients were omitted due to missing data, (n = 88). The total duration of follow-up was 379 person-years, and 53 deaths from SBA were observed. In Cox regression model with adjustment for sex, tumor stage, and age at operation, MSI tumors were associated with better disease-specific survival compared to MSS tumors (hazard ratio (HR), 0.111; 95% CI, 0.0292–0.419; P = 1.20x10^-3^). P-values for unadjusted log-rank tests are shown in the figure. (PDF) [file pgen.1007200.s009.pdf]

S1 Fig. Kaplan-Meier estimates of disease-specific survival according to a) MMR status, b) age at operation, c) stage, and d) sex. Eighteen patients were omitted due to missing data, (n=88). The total duration of follow-up was 379 person-years, and 53 deaths from SBA were observed. In Cox regression model with adjustment for sex, tumor stage, and age at operation, MSI tumors were associated with better disease-specific survival compared to MSS tumors (hazard ratio (HR), 0.111; 95% CI, 0.0292-0.419; P=1.20x10^{-3}). P-values for unadjusted log-rank tests are shown in the figure.

a)

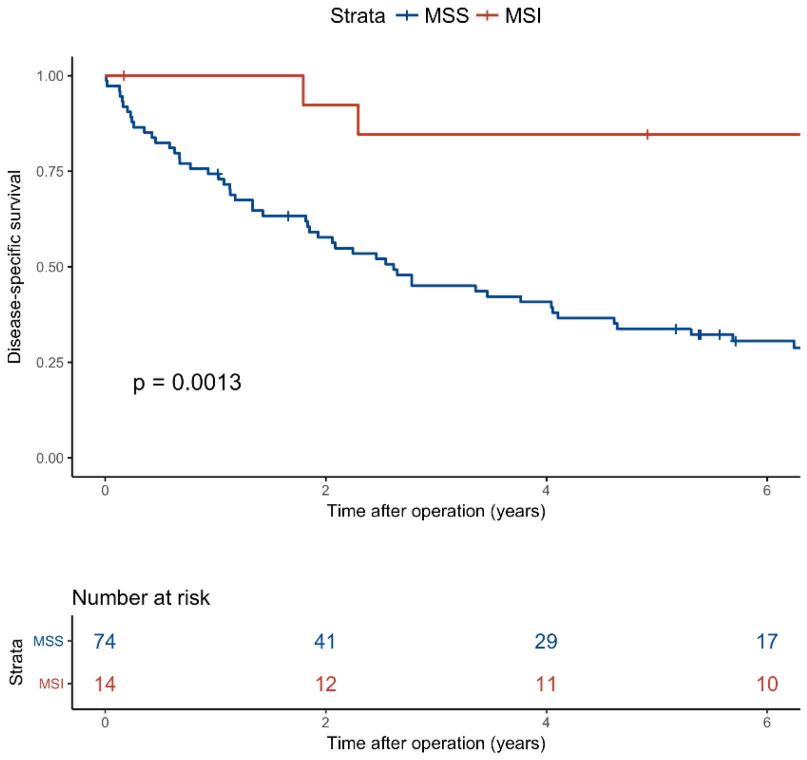

b)

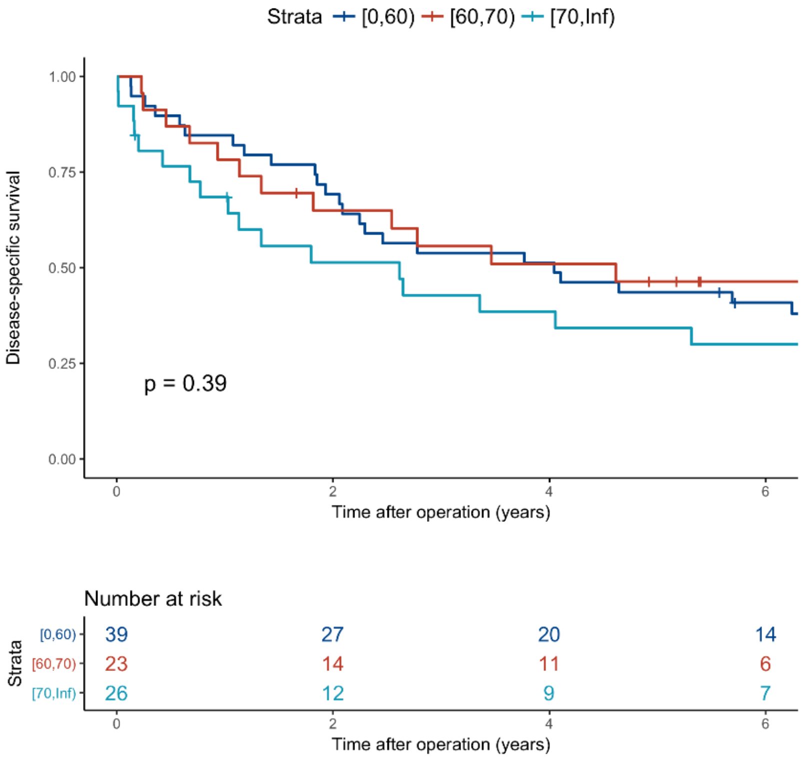

c)

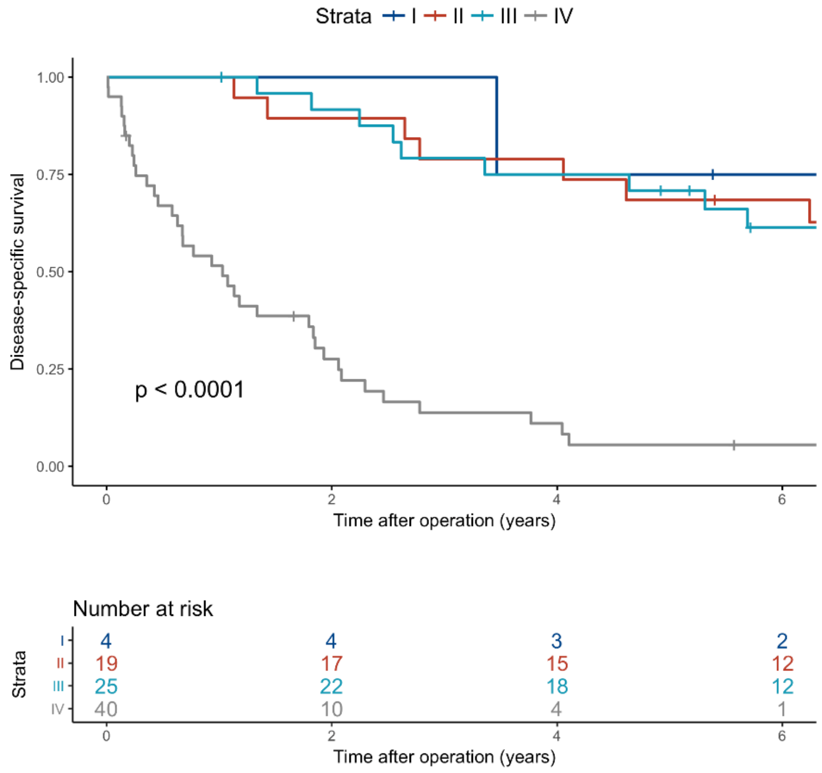
Strata I II III IV
Disease-specific survival
1.00
0.75
0.50
0.25
0.00
p < 0.0001
0
2
4
6
Time after operation (years)
Number at risk
Strata
I 4 4 3 2
II 19 17 15 12
III 25 22 18 12
IV 40 10 4 1
Time after operation (years)

d)

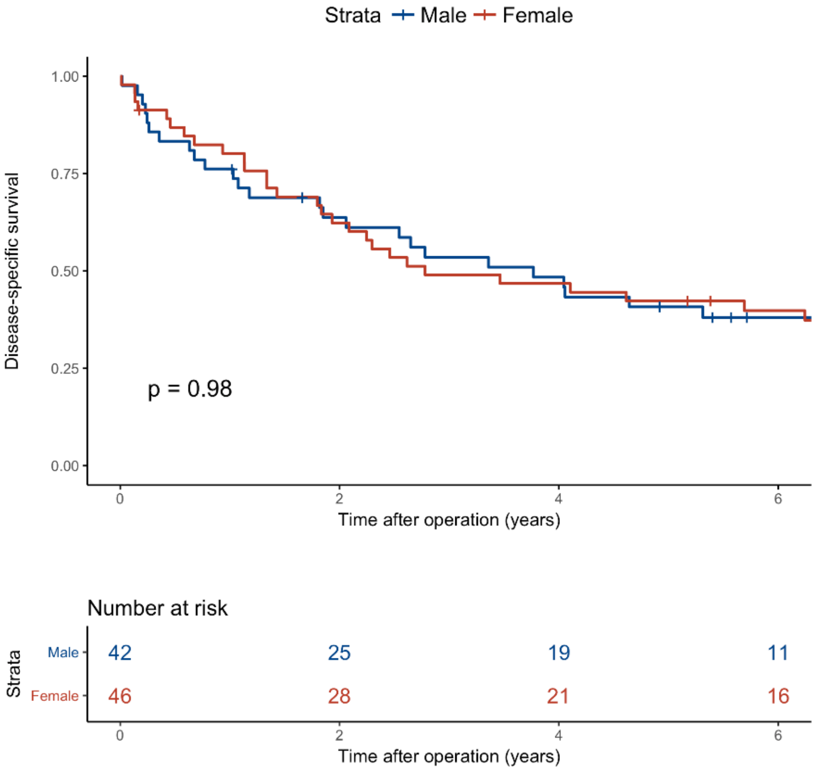
Strata Male Female
Disease-specific survival
1.00
0.75
0.50
0.25
0.00
p = 0.98
0
2
4
6
Time after operation (years)
Number at risk
Strata
Male 42 25 19 11
Female 46 28 21 16
Time after operation (years)
