# Supplementary figures and images for: Exome-wide somatic mutation characterization of small bowel adenocarcinoma
Source: PLoS Genet. 2018 Mar 9;14(3):e1007200. doi: 10.1371/journal.pgen.1007200 (PMC5871010; doi:10.1371/journal.pgen.1007200)

**S2 Fig. Somatic mutation prevalence.** The mutation burden in the whole set, n=106. Median value (red line) = 3.96.

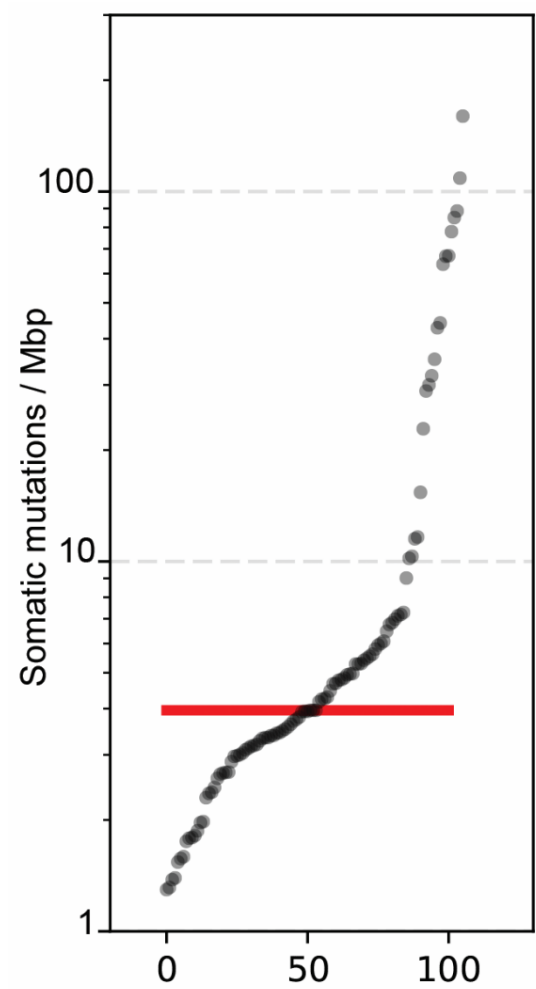

Supplement: S2 Fig — The mutation burden in the whole set, n = 106. Median value (red line) = 3.96. (PDF) [file pgen.1007200.s010.pdf]
