# Supplementary material for: Exome-wide somatic mutation characterization of small bowel adenocarcinoma
Source: PLoS Genet. 2018 Mar 9;14(3):e1007200. doi: 10.1371/journal.pgen.1007200 (PMC5871010; doi:10.1371/journal.pgen.1007200)
Supplement: S3 Fig — Genes highlighted in our study (the 25 highest-ranking genes in OncodriveFML and the ERBB-family genes) (red) and cancer census genes near visible AI peaks (purple) are depicted in the graphs. (PDF) [file pgen.1007200.s011.pdf]

S3 Fig. AI events in 91 MSS SBAs. Genes highlighted in our study (the 25 highest-ranking genes in OncodriveFML and the ERBB-family genes) (red) and cancer census genes near visible AI peaks (purple) are depicted in the graphs.

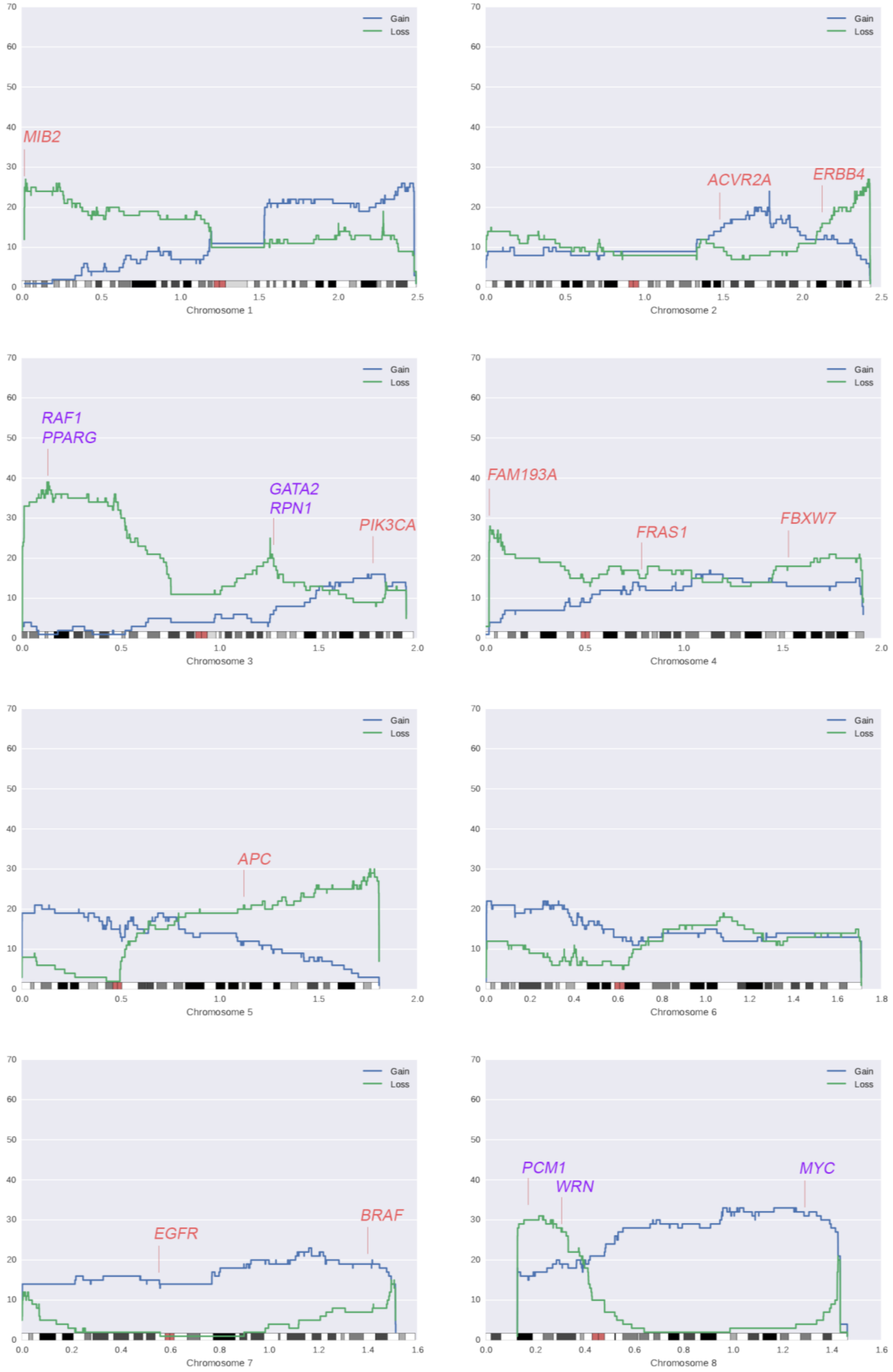

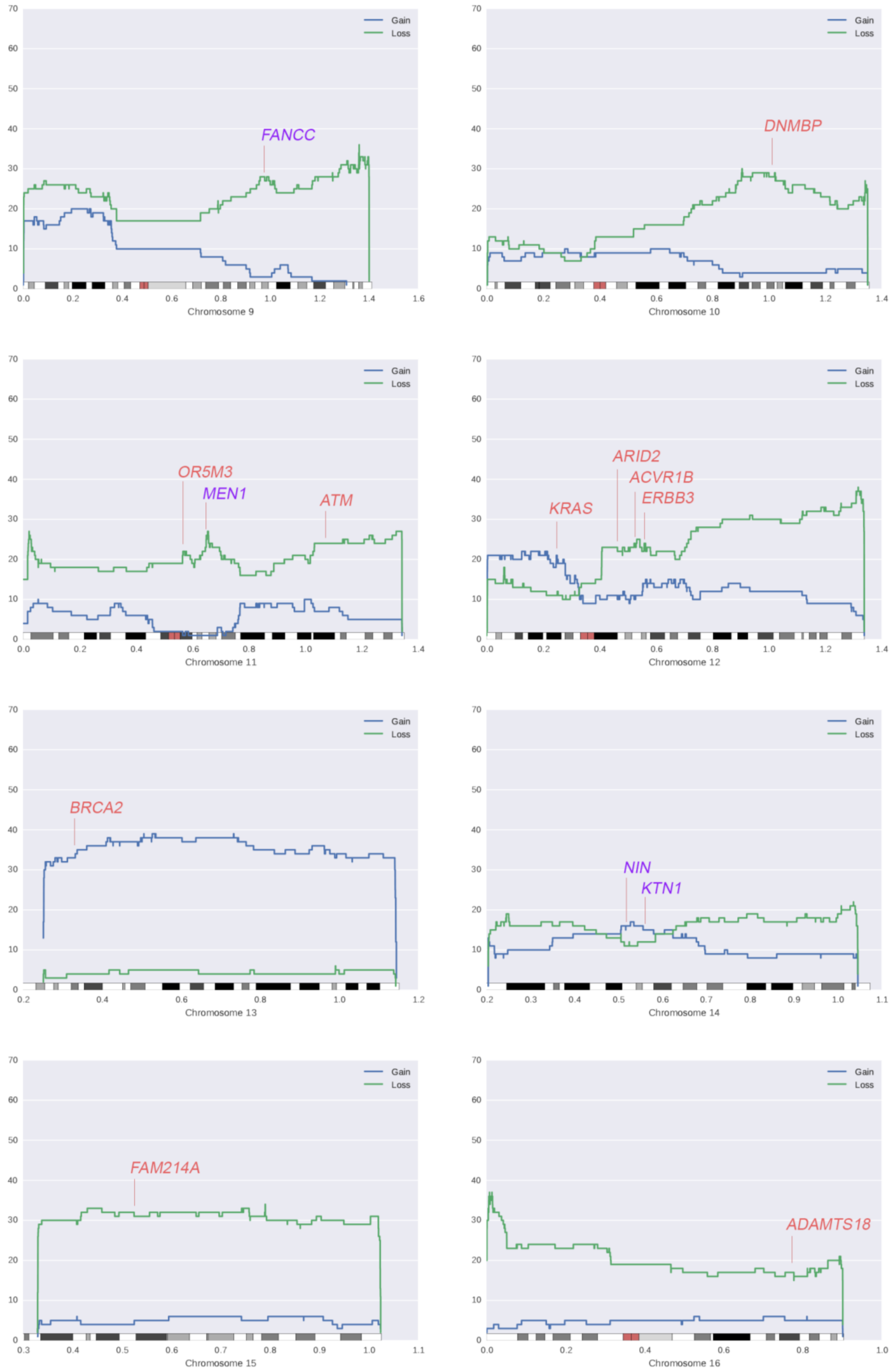
Gain
Loss
FANCC
Chromosome 9
DNMBP
Chromosome 10
OR5M3
MEN1
ATM
Chromosome 11
ARID2
ACVR1B
ERBB3
KRAS
Chromosome 12
BRCA2
Chromosome 13
NIN
KTN1
Chromosome 14
FAM214A
Chromosome 15
ADAMTS18
Chromosome 16

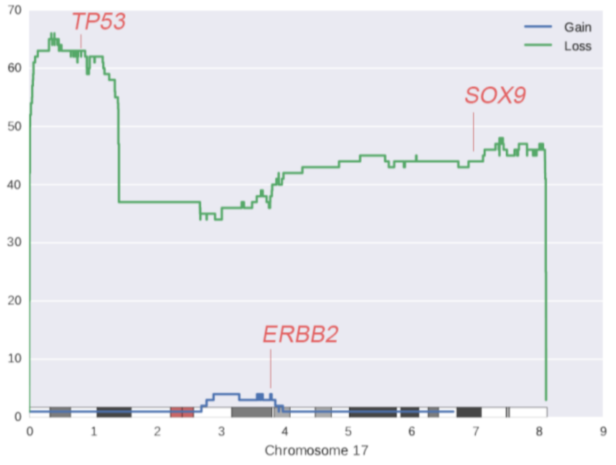
TP53
SOX9
ERBB2
Gain
Loss
Chromosome 17

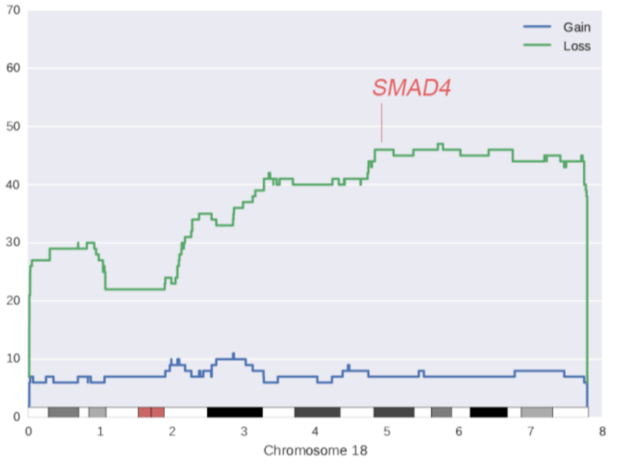
SMAD4
Gain
Loss
Chromosome 18

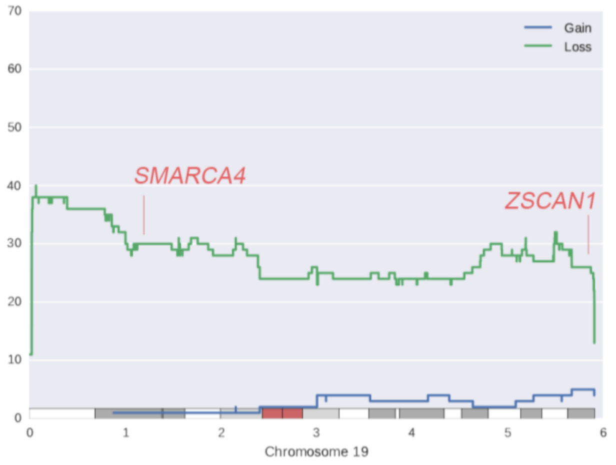
SMARCA4
ZSCAN1
Gain
Loss
Chromosome 19

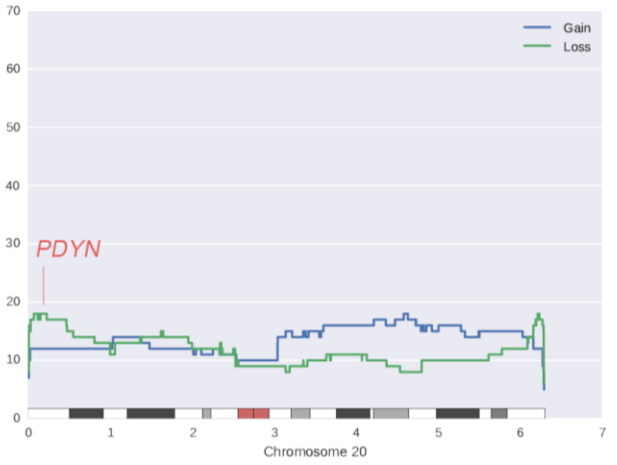
PDYN
Gain
Loss
Chromosome 20

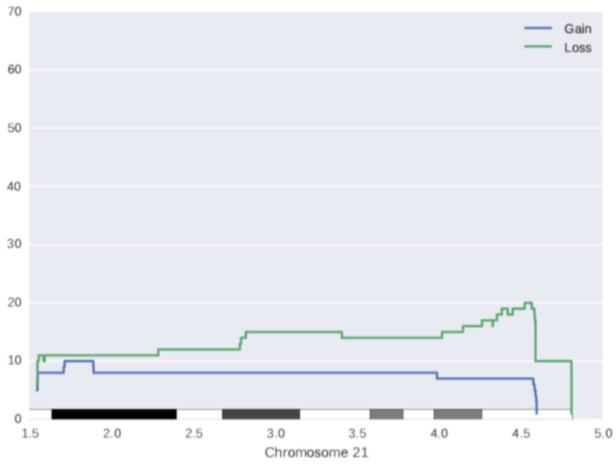
Gain
Loss
Chromosome 21

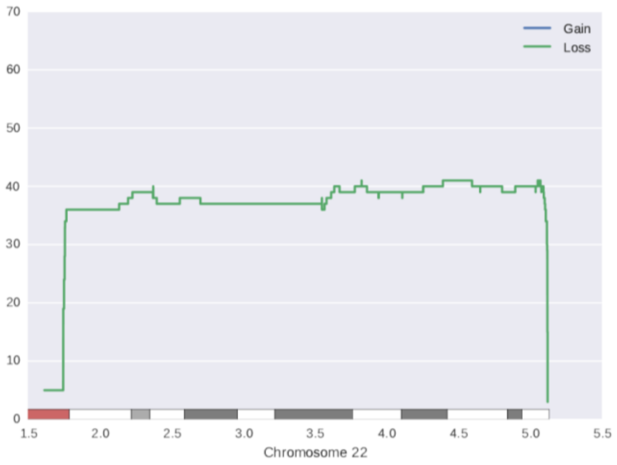
Gain
Loss
Chromosome 22

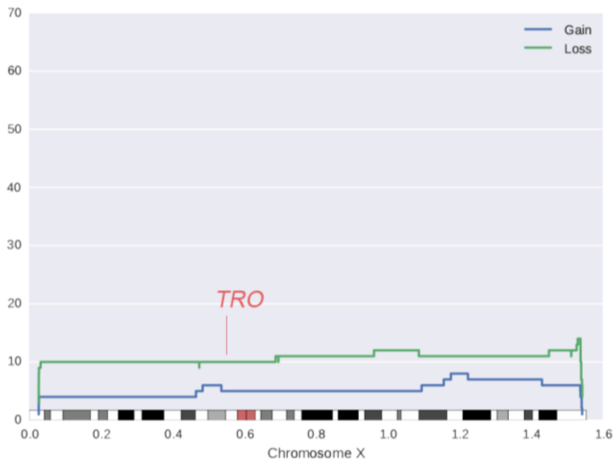
TRO
Gain
Loss
Chromosome X
