# Supplementary material for: Exome-wide somatic mutation characterization of small bowel adenocarcinoma
Source: PLoS Genet. 2018 Mar 9;14(3):e1007200. doi: 10.1371/journal.pgen.1007200 (PMC5871010; doi:10.1371/journal.pgen.1007200)

S4 Fig. Landscape of mutations and AI events in MSS SBAs. The figure includes the 25 highest-ranking genes in OncodriveFML and the ERBB-family genes. Different colors distinguish between the different types of AI events. Non-synonymous mutations are marked with a black dot.

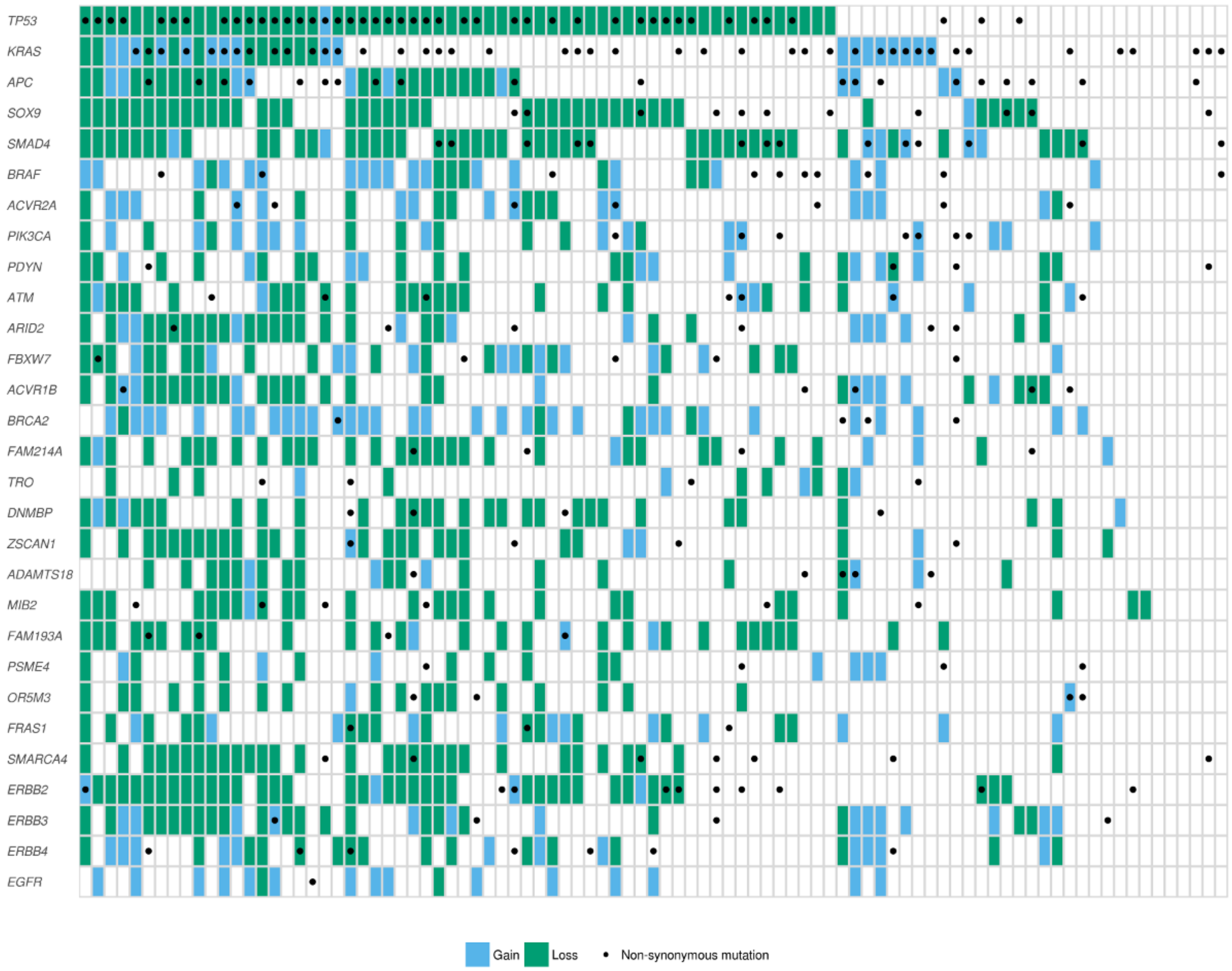

Supplement: S4 Fig — The figure includes the 25 highest-ranking genes in OncodriveFML and the ERBB-family genes. Different colors distinguish between the different types of AI events. Non-synonymous mutations are marked with a black dot. (PDF) [file pgen.1007200.s012.pdf]
