# Supplementary material for: Exome-wide somatic mutation characterization of small bowel adenocarcinoma
Source: PLoS Genet. 2018 Mar 9;14(3):e1007200. doi: 10.1371/journal.pgen.1007200 (PMC5871010; doi:10.1371/journal.pgen.1007200)

S5 Fig. AI events in the *ERBB2* and chromosome 17. Four tumors showed a strong localized amplification in *ERBB2*, of which two tumors harbored also *ERBB2* mutation (SIA82, V842I and SIA137, S310Y).

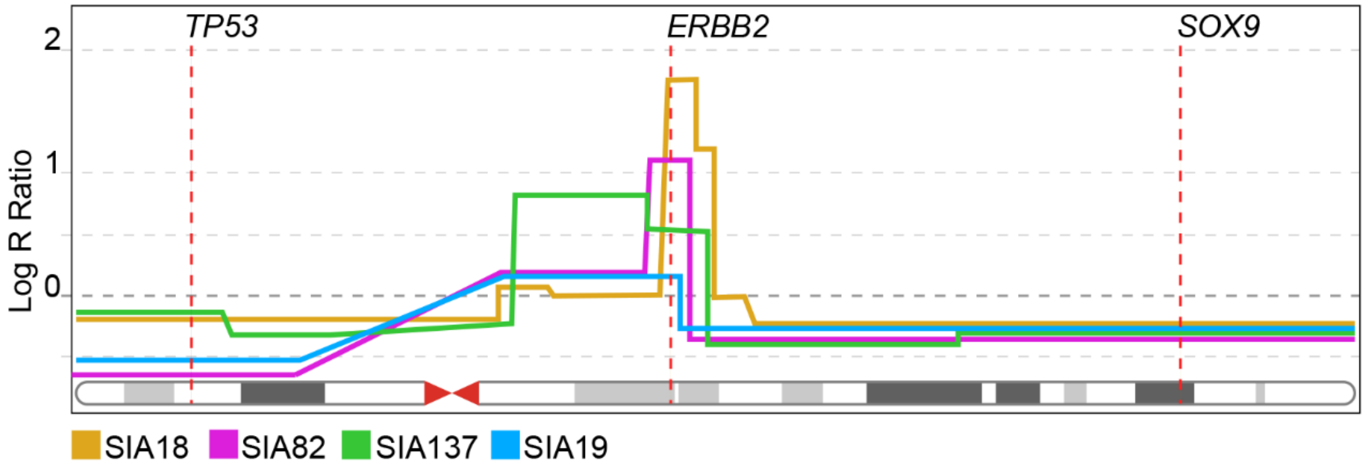

Supplement: S5 Fig — Four tumors showed a strong localized amplification in ERBB2, of which two tumors harbored also ERBB2 mutation (SIA82, V842I and SIA137, S310Y). (PDF) [file pgen.1007200.s013.pdf]
