# Supplementary material for: Exome-wide somatic mutation characterization of small bowel adenocarcinoma
Source: PLoS Genet. 2018 Mar 9;14(3):e1007200. doi: 10.1371/journal.pgen.1007200 (PMC5871010; doi:10.1371/journal.pgen.1007200)

S6 Fig. Comparison of signature 1A and age at diagnosis between tumors from different segments. Exposure to signature 1A was highest in jejunal tumors even though the median age at diagnosis was lower in patients with jejunal tumor compared to patients with duodenal or ileal tumors.

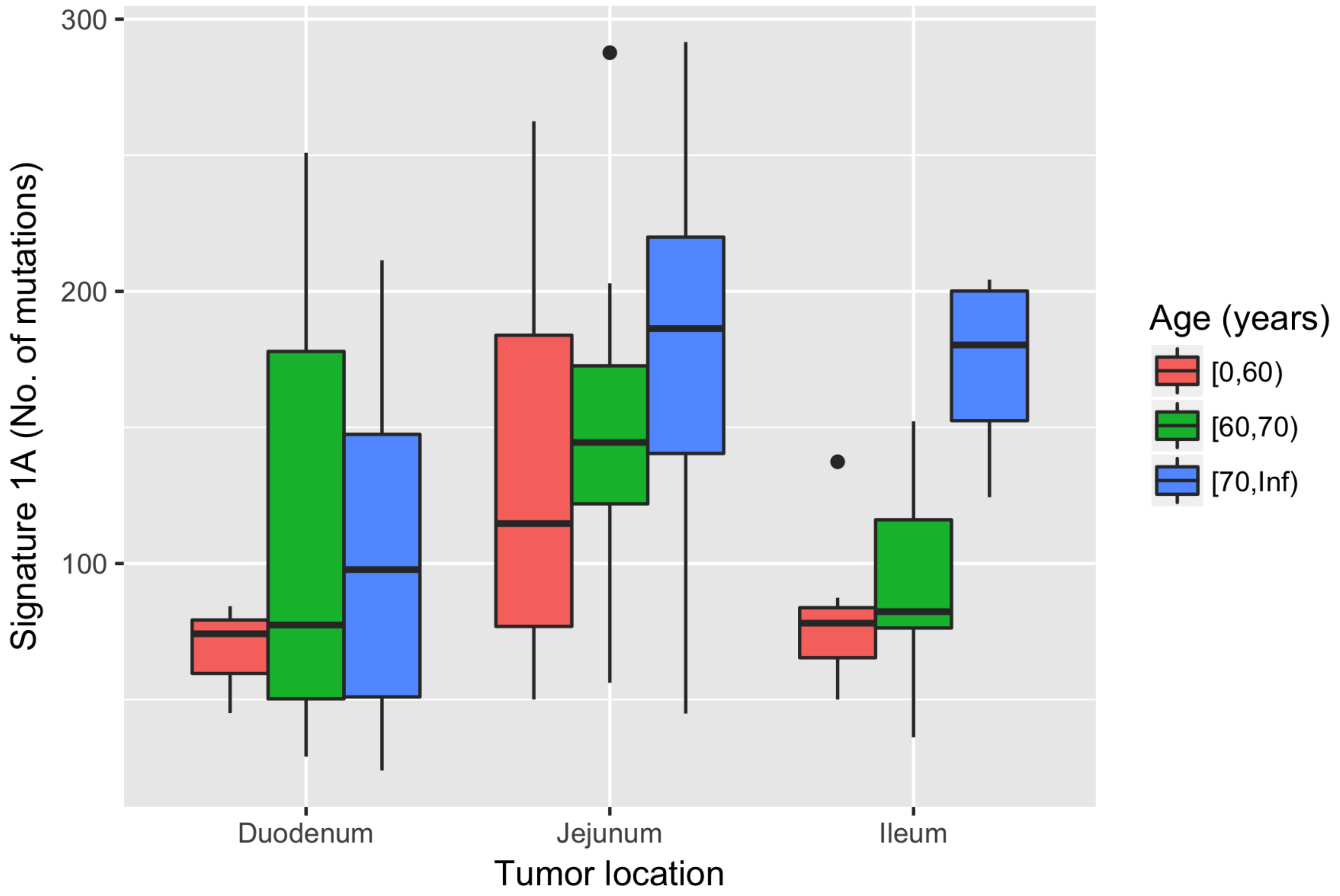

Supplement: S6 Fig — Exposure to signature 1A was highest in jejunal tumors even though the median age at diagnosis was lower in patients with jejunal tumor compared to patients with duodenal or ileal tumors. (PDF) [file pgen.1007200.s014.pdf]
