# Supplementary material for: Exome-wide somatic mutation characterization of small bowel adenocarcinoma
Source: PLoS Genet. 2018 Mar 9;14(3):e1007200. doi: 10.1371/journal.pgen.1007200 (PMC5871010; doi:10.1371/journal.pgen.1007200)

S7 Fig. Quantile-quantile plots for MSS (n=91) OncodriveFML analysis, (a) with all the genes included in the initial run and (b) after filtering the data to contain genes mutated in at least four samples.

a)

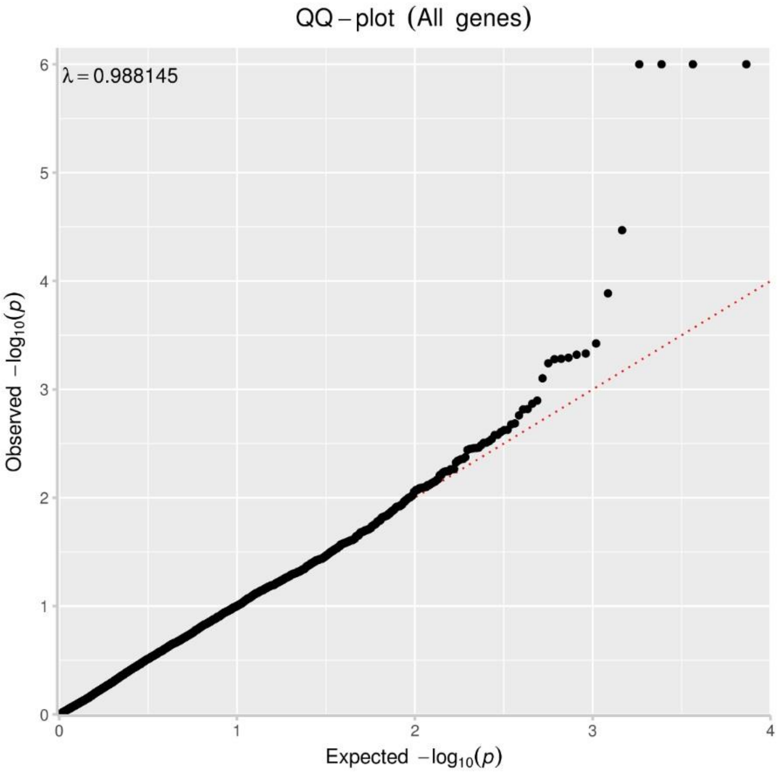

b)

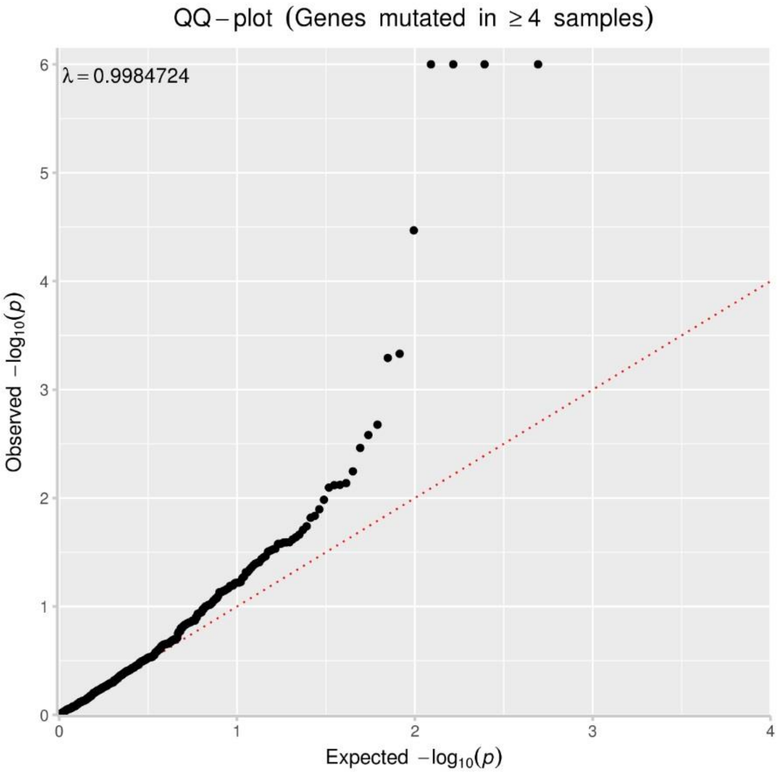

Supplement: S7 Fig — Quantile-quantile plots for MSS (n = 91) OncodriveFML analysis, (a) with all the genes included in the initial run and (b) after filtering the data to contain genes mutated in at least four samples. (PDF) [file pgen.1007200.s015.pdf]
